# Interfacial stability in single- and multi-junction perovskite solar cells

**DOI:** 10.1039/d6sc03324e

**Published:** 2026-06-29

**Authors:** Liangchen Qian, Bohong Chang, Danpeng Gao, Bo Li, Zonglong Zhu

**Affiliations:** a Department of Chemistry, City University of Hong Kong Hong Kong P. R. China zonglzhu@cityu.edu.hk; b School of Materials Science and Engineering, Central South University Changsha P. R. China boli72@csu.edu.cn; c Shenzhen Research Institute, City University of Hong Kong Shenzhen P. R. China

## Abstract

Perovskite photovoltaics offer high efficiencies and low costs, with single- and multi-junction cells showing immense future potential. Yet, their operational stability remains a key bottleneck that is increasingly rooted at interfaces. In this review, we analyze interfaces as metastable chemical microenvironments characterized by high defect densities, steep chemical potential gradients, localized electric fields, and mechanical stress. We establish a unified framework centered on the evolution of interfacial chemistry to compare degradation pathways in single-junction *versus* tandem cells. From these chemical insights, we distill actionable chemistry-to-function design rules, identify critical gaps in multi-junction interfacial stability, and outline the scalable engineering challenges that must be addressed to bridge laboratory success with commercial reality.

## Abstract 2

This review summarizes how ion migration, interfacial reactions, and stress/strain govern degradation in single- and multi-junction perovskite solar cells. Chemistry-to-function strategies for stable interfaces are discussed.

## Graphical abstract

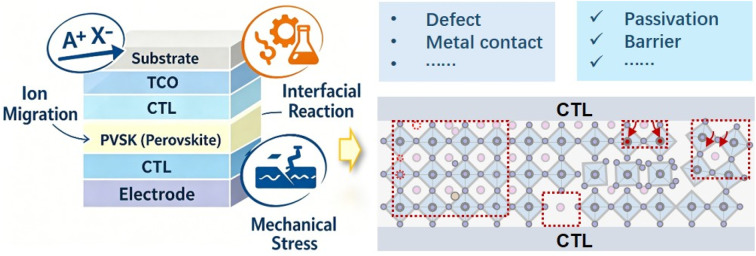

## 1 Introduction

**Fig. 1:**
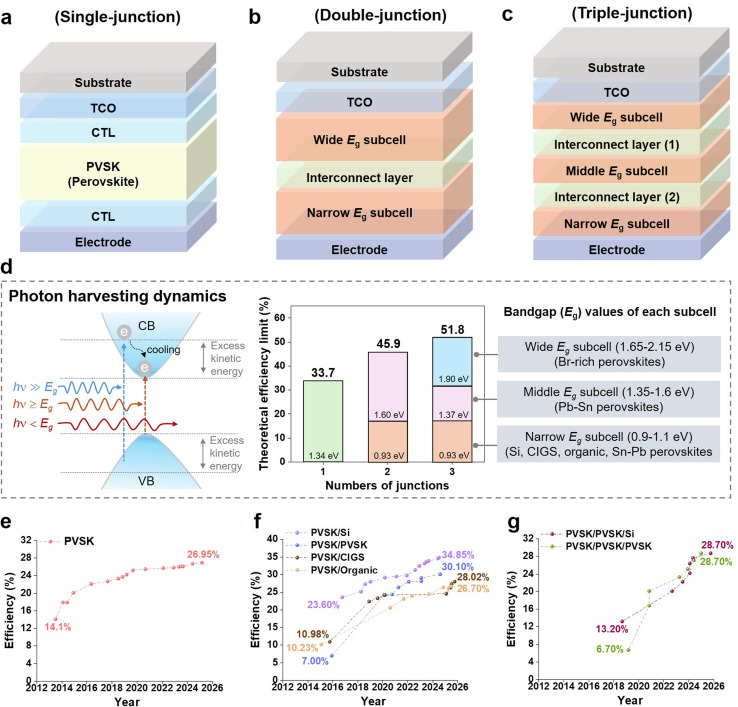
(a–c) Typical architectures of single-, double-, and triple-junction PSCs, where transparent conductive oxide (TCO), charge transport layer (CTL), wide *E*_g_, and narrow *E*_g_ represent the transparent conductive oxide, charge transport layer, wide-bandgap absorber, and narrow-bandgap absorber, respectively. In (b) and (c), each subcell consists of a perovskite layer sandwiched between two CTLs. (d) Photon harvesting dynamics and theoretical efficiency limits of PSCs, illustrating the corresponding Shockley–Queisser efficiency limits for single-junction (33.7%), double-junction (45.9%), and triple-junction (51.8%) devices. The narrow *E*_g_ subcells include silicon (Si), Cu(In,Ga)Se_2_ (CIGS), *etc.* (e–g) Certified and reported PCEs of single-junction and multi-junction PSCs. Certified data were obtained from the NREL Best Research-Cell Efficiency Chart, while non-certified data for multi-junction PSCs are extracted from ref. 21–42.

Metal halide perovskites have demonstrated exceptional potential for next-generation optoelectronics with landmark advances across solar cells, light-emitting diodes, photodetectors and lasers.^1^ Their widely tunable optical and electronic features, facile and low-cost processing, and favorable attributes such as long carrier diffusion lengths, strong light absorption, and notable defect tolerance have positioned perovskite solar cells (PSCs) among the most promising photovoltaic technologies.^2–6^ According to the device architecture and energy-harvesting strategy, PSCs can be broadly categorized into single-junction and multi-junction configurations. In single-junction devices (Fig. 1a), the perovskite absorber is sandwiched between an electron-transport layer (ETL) and a hole-transport layer (HTL). In contrast, multi-junction devices (Fig. 1b and c) employ monolithic series stacking of two or more subcells to achieve spectral management and electrical interconnection, thus enabling efficiencies beyond the Shockley–Queisser (S–Q) limit (Fig. 1d).^7–10^ Although the power conversion efficiencies (PCEs) of single-junction and multi-junction PSCs have exceeded 26% (Fig. 1e) and 34% (Fig. 1f and g), respectively,^11^ insufficient operational stability under realistic stressors such as heat, illumination, and humidity remains a central bottleneck for commercialization.^12–14^

From a chemical perspective, the stability challenge is not governed solely by the thermodynamic instability of the bulk perovskite; rather, it is more critically manifested as a destabilization of interfacial reaction chemistry and mass transport. Interfaces concurrently sustain cross-layer fluxes of ions, molecules, and electronic charge carriers. Local electric fields, photogenerated carriers, and temperature rises can reshape reaction pathways and kinetics, thereby activating acid–base and coordination interactions, ion-migration-driven changes in chemical potentials, interfacial redox and corrosion reactions, and reaction acceleration induced by light, heat, and electrical bias. These chemical processes ultimately translate into macroscopic failure signatures, including enhanced nonradiative recombination, energetic-level drift, contact degradation, and morphological evolution.^15–20^

Since the first report of perovskite-based inorganic-sensitized solar cells in 2009,^2^ PSCs have progressed from single-junction designs toward multi-junction architectures aimed at surpassing theoretical efficiency limits.^28,43^ Compared with single-junction PSCs, multi-junction devices reduce thermalization losses and enhance full-spectrum utilization by vertically stacking subcells with complementary bandgaps. A typical two-terminal tandem comprises a wide-bandgap (*ca.* 1.65–2.15 eV) Br-rich perovskite top cell paired with a narrow-bandgap (*ca.* 0.9–1.1 eV) bottom cell (*e.g.*, Si, Cu(In,Ga)Se_2_, or Sn–Pb perovskite), and can be further extended to higher-order multi-junction stacks.^44–47^ Correspondingly, the theoretical efficiency limit increases from 33.7% for single-junction cells to 45.9% for tandems and 51.8% for triple-junction devices (Fig. 1d).^48,49^ Along this trajectory, however, the number of interfaces, chemical heterogeneity, and accessible reaction channels increase markedly.^50–52^ More importantly, as device architectures evolve from single-junction to multi-junction, interfacial stability issues are no longer simply additive and the underlying reaction network is reconfigured with rising structural complexity. This makes interfacial stability a critical scientific and engineering priority, while multi-junction structures correspondingly impose stricter chemical demands on interfaces.

Consequently, this review places single-junction and multi-junction PSCs within a unified framework, using the underlying commonalities in interfacial chemistry across different device architectures as the central theme, thereby distinguishing it from existing reviews. By correlating interfacial reaction kinetics, ion/species transport, and macroscopic degradation signatures under diverse stress conditions, we systematically delineate degradation pathways and distill interfacial-chemistry design principles and structural strategies that jointly address efficiency and durability, providing verifiable stabilization approaches to bridge laboratory records and scalable, commercial perovskite photovoltaics.

## 2 Foundational principles of interfacial instability: a chemical perspective

**Fig. 2:**
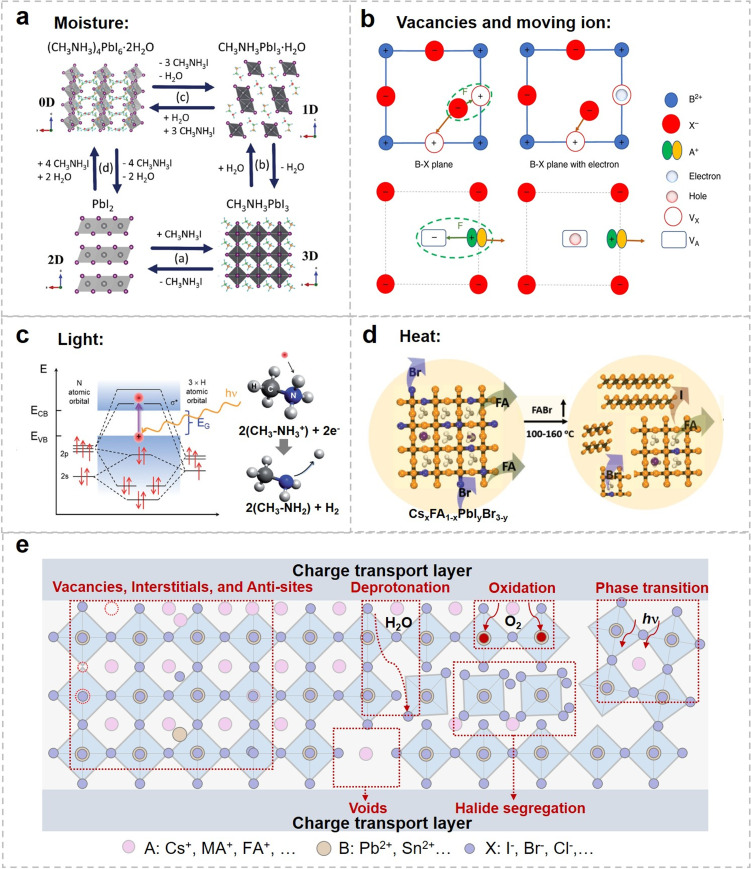
(a) Moisture-induced degradation of MAPbI_3_, involving reversible hydration and dehydration steps that, under prolonged moisture exposure or CH_3_NH_3_I removal, lead to irreversible phase separation, morphological changes, and PbI_2_ formation. Reproduced with permission from ref. 66, Copyright 2016, Wiley-VCH Verlag GmbH & Co. KGaA, Weinheim. (b) Schematic illustration of vacancy- and ion-migration induced degradation. In the B–X plane, excess electrons neutralize V_*x*_ to promote X^−^ migration, while in the A–X plane, excess holes neutralize V_a_ to facilitate A^+^ migration. Reproduced with permission from ref. 67, Copyright 2018, Springer Nature. (c) Light-induced degradation of the ammonium cation in perovskites, where photoexcited electrons captured by the antibonding orbital of ammonia leads to N–H bond cleavage and hydrogen atom abstraction from CH_3_NH_3_^+^. Reproduced with permission from ref. 68, Copyright 2017, Wiley-VCH Verlag GmbH & Co. KGaA, Weinheim. (d) Thermal degradation of the mixed cation perovskite Cs_*x*_FA_1−*x*_PbI_*y*_Br_3−*y*_. Reproduced with permission from ref. 69, Copyright 2018, Wiley-VCH Verlag GmbH & Co. KGaA, Weinheim. (e) Schematic representation of chemical degradation pathways in perovskites and PSCs, highlighting the influence of lattice defects (vacancies, interstitials, and anti-sites) and environment-induced processes such as void formation, deprotonation, oxidation, halide segregation, and phase transition.

In PSCs, external stressors (*e.g.*, humidity,^53–55^ illumination,^56–58^ heat,^59–61^ and oxygen^62–64^) are widely recognized accelerants of perovskite degradation, and their macroscopic effects have been thoroughly documented in dedicated stability articles.^65^ For example, as illustrated in Fig. 2a, MAPbI_3_ degradation proceeds *via* hydration–dehydration processes, inducing local phase separation and morphological changes that, under continuous moisture exposure or CH_3_NH_3_I removal, become irreversible and lead to PbI_2_ formation.^66^Fig. 2b suggests that excess charges originating from photo-generated carriers or carriers injected by current can neutralize ion vacancies and reduce coulombic drag associated with ion migration, leading to increased ion migration and decreased stability of PSCs.^67^ As shown in Fig. 2c, illumination induces excitations of electrons from the valence band to high-energy antibonding orbitals of the MA^+^ cation, thereby enabling bond dissociation and the release of H_2_.^68^ As shown in Fig. 2d, the mixed cation perovskite Cs_*x*_FA_1−*x*_PbI_*y*_Br_3−*y*_ exhibits preferential volatilization of bromide over iodide under thermal aging, resulting in the formation of decomposition products PbI_2_ and CsPbI_3−*z*_Br_*z*_.^69^ In Fig. 2e, the primary chemical degradation pathways in both single- and multi-junction PSCs originate from intrinsic lattice defects (*e.g.*, vacancies, interstitials, and anti-sites) and from environment-induced processes (*e.g.*, compositional deprotonation, oxidation, halide migration, and phase transitions).

While these environmental factors govern the rate at which degradation proceeds, the fundamental vulnerabilities originate from the interfaces themselves. Here we focus on the intrinsic chemical drivers at interfaces. The interfacial instability of PSCs does not arise from a single physical or chemical process; rather, it results from the interplay of multiple mechanisms, including chemical reactions and defect chemistry, interfacial mass transport and diffusion, and interfacial stress–strain effects.^70^ Specifically, chemical reactions can generate defects and alter the local stress state; the diffusion of ions or constituent species can, in turn, further accelerate reaction kinetics; and regions of stress concentration often become preferential sites for diffusion and reactions, thereby forming a mutually reinforcing degradation “cascade.” As device architectures evolve from single-junction to tandem configurations, the introduction of key functional layers, such as interconnecting recombination layers and transparent conductive electrodes, substantially broadens and complicates interfacial degradation pathways, rendering multijunction devices more sensitive to interfacial instability.^71^

From a thermodynamic perspective, the total Gibbs free energy of a device can be regarded as the sum of bulk free-energy contributions from each layer and interfacial free-energy contributions from each interface. The fundamental driving force for interfacial destabilization can thus be unified as the tendency of the system to reduce its total free energy: any process that lowers the total free energy, whether interfacial chemical reactions, mutual interdiffusion of components, or stress relaxation, is thermodynamically spontaneous.^72^ However, the rates of these spontaneous processes in practice are constrained by kinetic barriers, leading to complex time- and temperature-dependent interfacial degradation.

Here, we elucidate the fundamental principles and microscopic origins of interfacial instability from three perspectives: (i) chemical reactions and defect chemistry, (ii) interfacial mass transport and diffusion, and (iii) interfacial stress and strain. Understanding these principles provides essential guidance for designing stable interfacial layers (*e.g.*, passivation layers and stress-buffer layers) and for optimizing device architectures.

### 2.1 Instability induced by interfacial chemical reactions and defect chemistry

Chemical reactions between adjacent functional layers in a device are among the most direct origins of interfacial degradation. At heterogeneous material interfaces, the chemical potentials of the constituent species are typically discontinuous. The interfacial chemical-potential difference (Δ*µ*) constitutes the intrinsic driving force for interfacial reactions, driving the system to spontaneously evolve toward a lower Gibbs free energy.^73^ The thermodynamic spontaneity of an interfacial reaction is determined by the Gibbs free-energy change (Δ*G*_r_) of the reaction; when Δ*G*_r_ < 0, the reaction is thermodynamically spontaneous:

1 Δ*G*_r_ = Δ*H*_r_ − *T*Δ*S*_r_ = −*RT* ln *K*_eq_

where Δ*H*_r_ and Δ*S*_r_ denote the reaction enthalpy change and entropy change, respectively; *T* is the temperature; *R* is the gas constant; and *K*_eq_ is the equilibrium constant of the reaction.

**Fig. 3:**
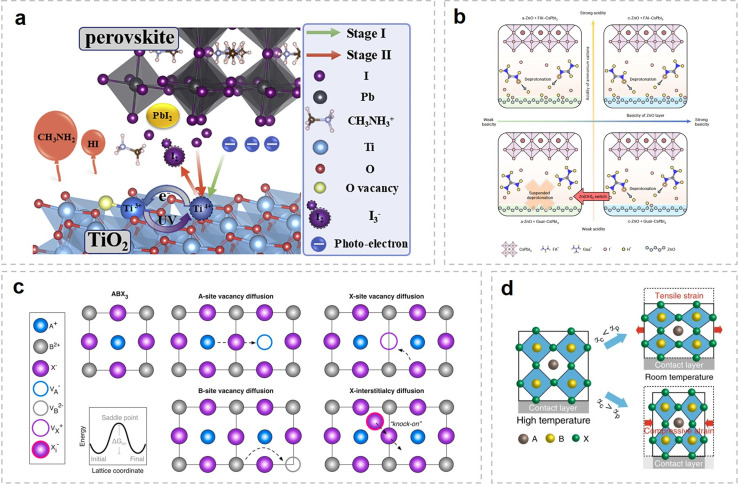
(a) UV-induced degradation pathway at the perovskite/ETL interface. Photoexcited electrons are captured by Ti^4+^–V_O_ states, reducing Ti^4+^ to Ti^3+^ and accelerating interfacial decomposition. Reproduced with permission from ref. 76. (b) Switchable interfacial deprotonation mechanism. The basic Zn(OH)_2_ intermediate facilitates proton abstraction from organic cations, while subsequent conversion to ZnO terminates the reaction. Reproduced with permission from ref. 80, Copyright 2024, Springer Nature. (c) Schematic of ion migration mediated by charged point defects in ABX_3_ perovskites. Vacancies and interstitials act as the primary transport vehicles for halide and cation diffusion. Reproduced with permission from ref. 84, Copyright 2018, American Chemical Society. (d) Origin of thermal-mismatch tensile strain. Upon cooling from annealing temperature, constrained contraction of the perovskite film on the substrate induces in-plane tensile strain. Reproduced with permission from ref. 85, Copyright 2020, Springer Nature.

Interfaces are intrinsically regions where lattice periodicity is disrupted. Under-coordinated atoms and dangling bonds further reduce the activation barriers for chemical reactions.^74^ However, whether interfacial reactions occur in practice and their specific reaction rates are governed by kinetic factors, primarily the activation energy, the interfacial transport rate of reactants, and the concentration of interfacial defects. Moreover, external environmental stimuli (*e.g.*, illumination, electric fields, humidity, and heat) can markedly exacerbate interfacial chemical instability.^75^ For instance, the energy of photogenerated charge carriers can help overcome kinetic barriers, enabling reactions that are suppressed in the dark to proceed under illumination. Interfacial electric fields can modulate reaction kinetics by altering defect formation energies and migration barriers. Moisture and oxygen can directly participate in degradation reactions, opening additional reaction pathways. If the reaction products are continuously removed or consumed, the equilibrium will be persistently driven to the right, resulting in sustained perovskite degradation at the interface. Fig. 3a provides a specific example of such photo-induced degradation at the perovskite/electron-transport-layer interface. Under continuous UV illumination, oxygen vacancies in TiO_2_ actively capture photogenerated electrons from the perovskite, driving the reduction of Ti^4+^–V_O_ to Ti^3+^–V_O_ states.^76^ This interfacial charge trapping not only promotes non-radiative recombination but also serves as a chemical precursor step that accelerates the decomposition of the perovskite absorber.

From a mechanistic standpoint, the primary factors that induce an imbalance in interfacial chemical potentials and thereby trigger interfacial destabilization are as follows.

Perovskites exhibit intrinsic chemical metastability, meaning that under specific conditions, the Gibbs free energy of their decomposition products can be lower than that of the perovskite phase. At interfaces, the effects of strain fields, defect density, and the local chemical environment can further reduce the kinetic barriers of the perovskite, making it highly susceptible to chemical reactions or phase transitions. For example, for MAPbI_3_, which exhibits a relatively low free energy of decomposition, even mild external perturbations can induce decomposition into MAI and PbI_2_. Likewise, the photoactive α-FAPbI_3_ phase is metastable at room temperature and readily transforms into the thermodynamically stable but non-photoactive δ-phase.^77^ In addition, the reaction barrier of perovskites at the interface can be strongly modulated by the chemical reactivity of adjacent layers.

Interfacial reactions between perovskite and functional layers are particularly representative of acid–base chemistry and proton-transfer reactions. The A-site organic cations in perovskites act as the conjugate acids of weak bases and can donate protons to basic species. In contrast, the surfaces of many commonly used inorganic transport layers are intrinsically basic: hydroxyl groups and oxygen-bridge sites on metal oxides can serve as basic oxygen sites, thereby triggering proton-transfer reactions. Such reactions can directly induce local collapse of the perovskite lattice at the interface, creating interfacial degradation regions. Moreover, halide ions (especially I^−^), exhibit substantial reducing character, whereas metal cations in certain transport layers (*e.g.*, Ni^3+^ in NiO_*x*_ and Mo^6+^ in MoO_*x*_) are oxidizing; together they form a redox couple at the interface.^78^ The proton-transfer reactivity at such interfaces is not static but can evolve with the chemical state of the transport layer itself. As illustrated in Fig. 3b, the interfacial deprotonation reaction can be “switched on” by the presence of a basic hydroxide intermediate (*e.g.*, Zn(OH)_2_) and subsequently “switched off” upon its conversion to a less basic oxide (*e.g.*, ZnO), highlighting how the dynamic surface chemistry of the adjacent layer governs the extent of organic cation deprotonation and associated lattice destabilization.^79^ Under illumination, iodide can be oxidized by photogenerated holes to iodine atoms or iodine radicals. These iodine radicals can recombine to form molecular iodine (I_2_), which can further oxidize organic cations or react with the transport-layer material.^80^

Interfacial defects tend to preferentially form and accumulate, especially under device operating conditions. The field-driven, directional migration of charged defects (predominantly ionic species) under an electric field underpins a range of dynamic instabilities in perovskite devices. The high concentration of defects enriched at interfaces not only directly induces electrical degradation, but also catalyzes and accelerates interfacial chemical reactions, giving rise to a degradation mechanism involving defect chemistry and interfacial reactions.^81^ In particular, the accumulation of iodide vacancies at interfaces lowers the local iodine chemical potential (*i.e.*, creates an iodine-deficient environment) and shifts the local thermodynamic equilibrium, rendering additional defect-mediated reactions thermodynamically accessible.^82^ For example, under extremely iodine-poor conditions, perovskites can undergo deep decomposition as follows:2
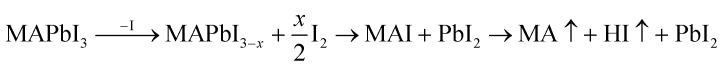


By contrast, at iodine-rich interfaces (on the side enriched in iodine interstitials), the high concentration of interstitial iodine atoms/ions readily leads to oxidative reactions with the transport layer, or promotes iodine loss, particularly under illumination, where I^−^ can be oxidized to I_2_. Iodine loss can lead to iodine doping or iodine contamination of the transport layer, thereby altering its electronic properties.^83^ For example, in perovskite/silicon tandem devices, iodine diffusion to the silicon surface can severely deteriorate the passivating interface of the a-Si : H/c-Si heterojunction, introducing recombination-active defect states within the silicon bandgap.

Moreover, during the fabrication of tandem devices, the deposition of each subsequent layer can potentially damage the underlying layers that have already been formed. When the top subcell is fabricated *via* solution processing, the solvent can infiltrate the interconnecting layer (ICL) or the surface of the bottom subcell, leading to dissolution or recrystallization of interfacial modification layers. Such process-induced interfacial defects establish an initial defect population that, during subsequent device operation, serves as nucleation sites for interfacial degradation reactions and as fast pathways for ion migration.

### 2.2 Interfacial instability induced by mass diffusion

The multilayer heterostructures of PSCs remain in a thermodynamically nonequilibrium state during operation, with pervasive chemical-potential gradients between functional layers and between the device and its environment. Mass transport and diffusion are spontaneous material-transport processes that arise in response to these gradients. Microscopically, they govern the evolution of interfacial composition; macroscopically, they control the rate and mode of device performance decay. Mass transport and diffusion primarily involve the spatial redistribution of atoms, ions, and molecules, and are therefore distinct from chemical reactions that entail bond breaking and re-formation. This comparatively “mild” migration process is nonetheless pivotal: it propagates local chemical perturbations throughout the device and accumulates transient microscopic damage into irreversible macroscopic degradation. Halide ions, organic cations, metal ions, and environmental molecules can all diffuse, although their diffusion coefficients, pathways, and driving forces differ, and collectively drive interfacial compositions away from their designed states, ultimately triggering cascade structural degradation and optoelectronic performance losses.^86^

Ion migration in perovskites plays a critical role in their instability. Unlike conventional inorganic covalent semiconductors (*e.g.*, Si and GaAs), ion migration in ionic lattices is a primary origin of interfacial instability in perovskite materials.^87^ Ions migrate through the lattice *via* point defects (vacancies or interstitials), and the diffusion kinetics typically follow an Arrhenius relationship:3
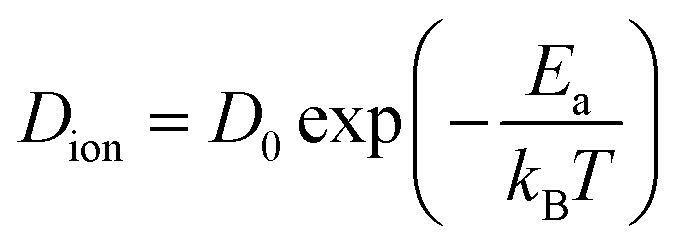
where *D*_ion_ is the ionic diffusion coefficient, *D*_0_ is the prefactor (related to the lattice vibrational frequency and the ionic hopping distance), and *E*_a_ is the activation energy for ion migration.^88,89^ The microscopic mechanisms underlying this defect-mediated transport are illustrated in Fig. 3c. Halide ions (X^−^) and A-site cations migrate predominantly *via* hopping between adjacent vacancy sites, while halide interstitials can also contribute to mass transport under certain stoichiometric conditions. These point-defect-mediated pathways constitute the elementary steps governing the long-range ionic redistribution that drives interfacial compositional evolution.^84^

Under operating conditions, the driving forces for ion migration include not only concentration gradients, but also electric-field drift and chemical-potential gradients. This ion-transport behavior, jointly driven by concentration gradients and the built-in electric field, can be fully described by the Nernst–Planck equation for the ionic flux (*J*_*i*_):4
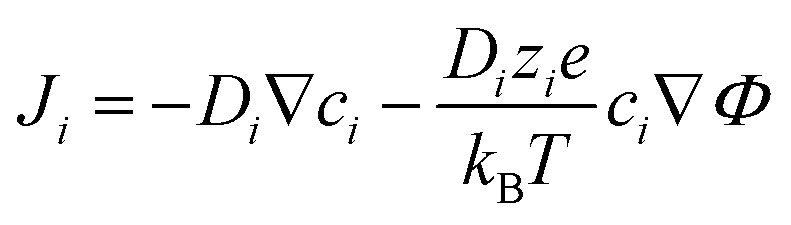
where *D*_*i*_ is the diffusion coefficient, ∇*c*_*i*_ is the concentration gradient, *z*_*i*_ is the ionic charge number, *e* is the elementary charge, and ∇*Φ* is the electric-potential gradient.^90^ The first term corresponds to Fickian diffusion driven by the concentration gradient, whereas the second term represents electromigration driven by the electric field.^91^ Owing to the built-in electric field at interfaces, the electromigration term is often dominant.

Halide ions are the most mobile species in perovskites, whereas the intrinsic diffusion of Pb^2+^ at typical operating temperatures is generally negligible. Ion migration drives ionic species to accumulate at interfaces, forming an ion-accumulation layer. Because cations and anions preferentially pile up at opposite interfaces, a space-charge region develops, which can partially screen the built-in electric field. This screening effect is widely regarded as a primary origin of scan-direction-dependent current–voltage (*J*–*V*) hysteresis.^92^ It is also a precursor to long-term degradation at perovskite/transport-layer interfaces, as it enriches reactants locally and thereby facilitates subsequent interfacial chemical reactions.

Cross-interface elemental diffusion occurs when atoms or molecules exchange across adjacent functional layers by traversing the interface under a chemical-potential difference. Such cross-interface ionic diffusion not only disrupts the perovskite stoichiometry, but can also deactivate doping in charge-transport layers and corrode metal electrodes. For example, iodine species migrating to the transport-layer/metal-electrode (Ag) interface can undergo thermodynamically spontaneous reactions to form metal iodides (AgI).^93^ Outward diffusion of organic cations can lead to A-site deficiency at the interface and precipitation of a PbI_2_ phase, thereby triggering interfacial instability. In tandem devices, the ICL can act as an “ion reservoir,” where migrated halides either react with the TCO or accumulate within the layer. In particular, ultrathin Au layers can corrode under iodine-rich conditions and illumination, forming compounds such as AuI_3_. Moreover, diffusion of In and Sn from the ICLs into the perovskite, as well as diffusion of dopants (*e.g.*, P and B) from silicon-based subcells, can also contribute to instability.^94^

### 2.3 Interfacial-stress-induced instability

PSCs are multilayer stacks composed of materials with markedly different mechanical properties. Halide perovskites feature soft ionic lattices; organic transport layers exhibit viscoelasticity and glass-transition behavior; inorganic oxide functional layers are hard and brittle; and metal electrodes are ductile. Under thermal cycling or photothermal heating, interfacial stress can accumulate, triggering microcracks, interlayer delamination, or phase transformations. In single-junction devices, stress is dominated by mismatch between the substrate and the perovskite layer.^95^ In contrast, tandem solar cells comprise a complex multilayer stack with more than ten nanometer-scale thin films, resulting in highly nonuniform stress gradients that can be transmitted and amplified across the stack. In particular, the ICL is typically rigid and inorganic; when sandwiched between relatively compliant perovskite/organic layers, it creates pronounced mechanical-impedance mismatch. During thermal cycling, strain localization at the ICL/perovskite interface can be far more severe than in single-junction devices, making it a critical weak point for mechanical fatigue and delamination.^96,97^ Here, we focus on two ubiquitous classes of interfacial stress/strain, intrinsic lattice/structural mismatch and film-growth-induced stress, as well as thermally induced mismatch strain, and further analyze the effects among stress, diffusion, and interfacial reactions.

Intrinsic lattice/structural mismatch arises from crystallographic incompatibility between adjacent layers, fundamentally reflecting differences in bonding characteristics and atomic arrangements, while film-growth-induced stress adds an additional source of interfacial strain. When two crystals form an interface, disparities in lattice constants hinder coherent lattice matching; moreover, mismatched crystal symmetry across the interface is another important source of structural mismatch. Film-growth-induced stress refers to residual stress that becomes kinetically “frozen” within the film during deposition and crystallization, and its origin strongly depends on the growth route. In solution processing, stress can be introduced by nucleation and island coalescence, volume shrinkage associated with solvent evaporation and precursor-to-perovskite conversion, and stress evolution during grain growth and Ostwald ripening.^98,99^ In vapor deposition, evaporated layers often form with relatively low adatom kinetic energy, which can yield porous, insufficiently densified films and thus tensile stress. In magnetron sputtering, the bombardment by energetic particles densifies the substrate surface and the growing film (“atomic peening”), ultimately leading to compressive stress.

Thermally induced mismatch strain refers to the interfacial strain generated by temperature variations due to mismatched coefficients of thermal expansion between adjacent layers. Because the functional layers differ in lattice constants and coefficients of thermal expansion, pronounced residual stresses can develop at interfaces during post-annealing cooling or during thermally driven cycling associated with day–night temperature fluctuations. The thermal stress arising from thermal-expansion mismatch (*σ*_th_) can be approximately estimated from:5
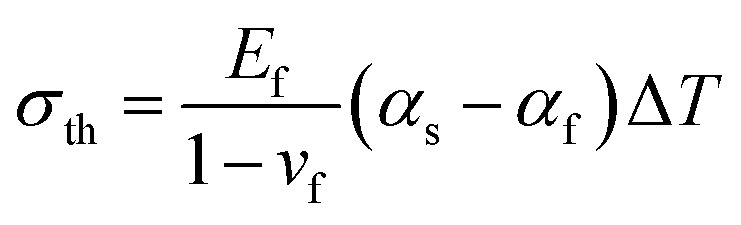
where *E*_f_ and *v*_f_ are the Young's modulus and Poisson's ratio of the film, respectively; *α*_s_ and *α*_f_ are the coefficients of thermal expansion (CTEs) of the substrate and the film, respectively; Δ*T* is the temperature change.^100^Fig. 3d schematically depicts the physical origin of such thermally induced strain. When a perovskite film is annealed at an elevated temperature and subsequently cooled on a substrate with a smaller coefficient of thermal expansion, the differential contraction is constrained by the interfacial adhesion, resulting in the accumulation of in-plane tensile strain within the perovskite layer. This residual strain field, concentrated near the interface, provides an additional driving force for defect migration and interfacial debonding during device operation.^85^

The effects among stress, diffusion, and interfacial reactions mean that these processes are not independent but instead form a positive-feedback loop, where stress gradients modulate mass transport and diffusion in turn affects the stress field. Stress gradients provide an additional driving force for mass transport and thus modulate diffusion: tensile stress increases the atomic chemical potential and promotes outward diffusion, whereas compressive stress lowers the chemical potential and favors species accumulation. Interfacial tensile stress induced by thermal mismatch can accelerate the outward diffusion of organic cations and halides, leading to faster interfacial compositional loss. In multilayer devices, stress gradients are typically steepest near interfaces, making stress-driven diffusion particularly pronounced. Stress can also alter reaction equilibria and kinetics. For interfacial reactions accompanied by volume expansion, tensile stress tends to facilitate reaction progression.^101^ Collectively, these effects establish a positive-feedback cycle of “stress-driven diffusion, interfacial compositional change, accelerated chemical reactions, reaction-induced volume change, and renewed stress generation.”

Taken together, the three perspectives outlined above, interfacial chemical reactions and defect chemistry, mass diffusion, and interfacial stress, do not operate in isolation but form a mutually reinforcing degradation cascade. For instance, halide oxidation generates iodine radicals and molecular iodine, which not only corrode adjacent transport layers but also introduce lattice strain and vacancy defects near the interface. These defects, in turn, lower the activation barriers for ion migration, accelerating the outward diffusion of iodide and organic cations, which further shifts the local chemical equilibrium and amplifies interfacial stress. This network underpins the progressive and often self-amplifying nature of interfacial degradation in PSCs, illustrating why stabilizing interfaces requires simultaneous management of chemical reactivity, ion transport, and mechanical strain.

## 3 Interfacial degradation and mitigation strategies in single- and multi-junction PSCs

As discussed above, the stability of single- and multi-junction PSCs remains far from commercialization due to complex interfacial interactions between perovskite materials and device architectures. Therefore, it is essential to develop strategies at both the material and device scales to mitigate chemical degradation. Several representative methods are summarized in Table 1.

**Table 1:** Summary of reported advanced stabilization strategies for single-junction PSCs^a^

Approaches	Device structure	PCE (%)	Operational stability	Ref.
PVSK/ETL	n–i–p	24.23%	*T*_90_, 6000 h	102
PVSK/ETL	p–i–n	25.1%	*T*_96_, 2000 h	103
PVSK/ETL	p–i–n	25.40%	*T*_97_, 3670 h	104
PVSK/HTL	n–i–p	25.6%	*T*_81.7_, 1000 h	105
PVSK/ETL	p–i–n	26.15%	*T*_95_, 1200 h	106
PVSK/ETL	p–i–n	25.49%	*T*_87_, 2400 h	107
PVSK/ETL	p–i–n	25.20%	*T*_97_, 800 h	108
PVSK/ETL	p–i–n	25.10%	*T*_95_, 2000 h	109
PVSK/ETL	p–i–n	26.1%	*T*_98_, 2100 h	110
HTL/PVSK	p–i–n	25.6%	*T*_90_, 1000 h	52
HTL/PVSK	p–i–n	26.72%	*T*_97.2_, 2500 h	111
HTL/PVSK	p–i–n	25.05%	*T*_97.5_, 1600 h	112
HTL/PVSK	p–i–n	23.48%	*T*_95.4_, 1960 h	113
HTL/PVSK	p–i–n	26.2%	*T*_96_, 1200 h	114
HTL/PVSK	p–i–n	22.50%	No loss, 2000 h	115
HTL/PVSK	p–i–n	25.60%	*T*_90_, 1200 h	116
HTL/PVSK	p–i–n	25.56%	*T*_98_, 1000 h	117
HTL/PVSK	n–i–p	23.60%	*T*_98.7_, 1000 h	118
Additive (PVSK)	n–i–p	25.03%	*T*_99.98_, 969 h	119
Additive (PVSK)	n–i–p	26.41%	*T*_95_, 2000 h	120
Additive (PVSK)	n–i–p	25.03%	*T*_99.98_, 969 h	119
Additive (HTL)	n–i–p	25.56%	*T*_85_, 500 h	121
Additive (HTL)	n–i–p	25.02%	*T*_99_, 180 h	122
Additive (HTL)	n–i–p	26.2%	*T*_80_, 3000 h	123
Additive (HTL)	n–i–p	26.1%	*T*_95_, 1200 h	124
SAM	p–i–n	26.08%	*T*_98_, 1000 h	125
SAM	p–i–n	22.83%	*T*_90_, 1000 h	126
2D/3D structure	p–i–n	26.04%	*T*_96.7_, 1500 h	127
2D/3D structure	n–i–p	24.6%	*T*_85_, 1250 h	128
2D/3D structure	p–i–n	24.10%	*T*_97_, 1000 h	129
2D/3D structure	p–i–n	24.30%	*T*_95_, 500 h	130
2D/3D structure	p–i–n	26.7%	*T*_94_, 2000 h	131
2D/3D structure	p–i–n	24.30%	*T*_95_, 500 h	130
2D/3D structure	p–i–n	24.09%	*T*_85_, 1560 h	132
2D/3D structure	p–i–n	24.10%	*T*_90_, 1500 h	133
2D/3D structure	n–i–p	21.20%	*T*_90_, 4390 h	134

a The specified value (*e.g. T*_90_, 6000 h) for operational stability represents the device lifetime, defined as the time required for the efficiency to decrease to 90% of its initial value under continuous maximum power point tracking.

For single-junction PSCs, the perovskite/transport layer interface and the top metal electrode interface govern device failure. In multi-junction architectures, additional chemical complexity arises from the narrow-bandgap Sn–Pb perovskite subcell and the ICL have received much attention from researchers. The following subsections deconstruct these interfacial degradation pathways and review corresponding chemical mitigation strategies.

### 3.1 Two key interface and top metal contact degradation processes in single-junction PSCs

In single-junction PSCs, interfacial chemical instability primarily shows at two distinct frontiers. At the perovskite/transport layer interface, intrinsic defects and coordination chemistry with transport layer functional groups drive non-radiative recombination and phase decomposition. At the top metal electrode interface, electrochemical corrosion triggered by migrated halide ions directly causes contact degradation and device failure.^119,135–137^ In Section 3.1.1, we address the buried interface, reviewing strategies such as molecular coordination, interface engineering, and ion locking to passivate defects and stabilize the interphase. In Section 3.1.2, we focus on the top metal contact, where a halide-induced electrochemical cycle is mitigated by inert electrodes, barrier layers, or encapsulation.

#### 3.1.1 Coordination chemistry and defect passivation in the perovskite/ETL or HTL interface

At the perovskite/carrier transport layer (ETL or HTL) interface, local charge accumulation arises from strong Lewis acid–base sites formed by undercoordinated Pb^2+^ ions, halide vacancies, and A-site vacancies. This charge buildup constitutes the molecular origin of interfacial nonradiative recombination and chemical degradation. To address these defects through targeted chemical modulation, researchers have developed a variety of coordination strategies, ranging from bidentate bridging to multipoint chelation and electronic state engineering, all of which enhance interfacial stability from distinct chemical perspectives.

**Fig. 4:**
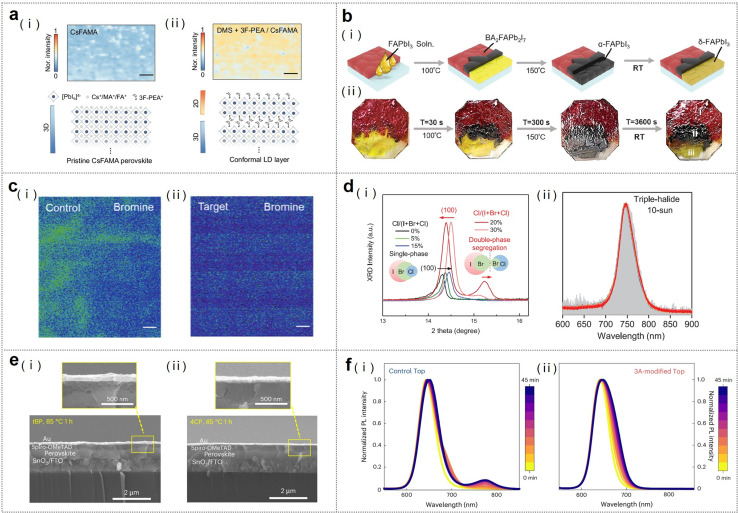
(a) Photoluminescence (PL) mapping of (i) pristine CsFAMA perovskite and (ii) DMS-modulated 3F-PEA/CsFAMA heterojunction films at 560 nm, corresponding to the *n* = 2 layer. Scale bar: 5 µm. The ellipsis indicates the continuity of the 3D structure. Reproduced with permission from ref. 131, Copyright 2025, Springer Nature. (b) Schematics of the templated FAPbI_3_ drop-coating process. (i) FAPbI_3_ precursor solution was dropped onto a glass substrate with BA_2_FAPb_2_I_7_ crystals. Upon heating, δ-FAPbI_3_ on BA_2_FAPb_2_I_7_ converted to α-FAPbI_3_ faster than on bare glass, while in air, α-FAPbI_3_ on bare glass reverted to δ-FAPbI_3_ before that on BA_2_FAPb_2_I_7_. (ii) Photographs showing three regions: BA_2_FAPb_2_I_7_ without FAPbI_3_, BA_2_FAPb_2_I_7_ under FAPbI_3_, and FAPbI_3_ on bare glass. Reproduced with permission from ref. 129, Copyright 2024, American Association for the Advancement of Science. (c) Time-of-flight secondary ion mass spectrometry (TOF-SIMS) mapping of bromine for control and target (TAACl-modified) perovskite films. Scale bar: 10 µm. Reproduced with permission from ref. 143, copyright 2025, Wiley-VCH Verlag GmbH & Co. KGaA, Weinheim. (d) (i) XRD (100) peak shifting and splitting indicate the transition from a single-phase triple-halide alloy to double-phase segregation with increasing Cl content. (ii) PL spectra of 1.67 eV triple-halide perovskites under 10-sun illumination. Reproduced with permission from ref. 144, Copyright 2020, American Association for the Advancement of Science. (e) Cross-sectional SEM images of single-junction PSCs using (i) tBP and (ii) 4CP additives after thermal aging at 85 °C for 1 hour. Reproduced with permission from ref. 123, Copyright 2025, Springer Nature. (f) Time-dependent PL spectra of (i) control and (ii) 3A-modified Cs_0.4_FA_0.6_PbBr_1.7_I_1.3_ perovskite aged under 1-sun illumination. Reproduced with permission from ref. 31, Copyright 2025, Springer Nature.

Using bidentate Lewis bases to bridge the interface is an effective strategy to strengthen both the mechanical and chemical stability of buried interfaces. For example, Li *et al.* employed the 1,3-bis(diphenylphosphino)propane (DPPP), which contains two phosphorus atoms that strongly coordinate with undercoordinated Pb^2+^*via* P–Pb bonds. Compared to theoretically stronger Lewis bases such as nitrogen, sulfur, and oxygen, phosphorus coordination exhibits higher binding energy due to the formation of a more stable tetrahedral geometry.^135^ This structural preference points to a refined screening principle for Lewis bases, the efficacy of which is corroborated by the significant suppression of halide-vacancy-associated photodecomposition following DPPP treatment. While the strong chelation of bidentate phosphines statically heals defect sites, the introduction of soft Lewis bases during dynamic heterojunction assembly presents a complementary, kinetics-driven pathway to mitigate recombination losses. As shown in Fig. 4a, introducing dimethyl sulfide (DMS), a soft Lewis base with high donor number and low dielectric constant, during the deposition of 3-fluoro-phenethylammonium (3F-PEA)/CsFAMA heterojunctions yields a spatially uniform photoluminescence profile. This uniformity reflects the role of soft–soft coordination between sulfur and Pb^2+^ in steering the formation of a conformal, defect-minimized interface, thereby suppressing non-radiative recombination.^131^ This approach underscores the utility of soft-base engineering in realizing conformal heterojunctions with minimal interfacial loss.

Beyond bidentate coordination, multipoint cooperative strategies further expand the chemical control over interfacial defects. Nie *et al.* reported a complex capable of simultaneously coordinating four neighboring Pb^2+^ ions within a single perovskite lattice unit through two selenium and two chlorine atoms. The chlorine atoms can directly occupy existing iodine vacancies due to favorable binding energies, while three NH–Cl hydrogen bonds in the ligand form an extended network with FA^+^ cations, further stabilizing the interface.^119^ This represents a spatially extended evolution of the Lewis-base passivation concept, wherein multipoint anchoring secures the lattice against degradation in a concerted manner.

In addition to coordination saturation, modulation of interfacial electronic states *via* Lewis bases offers another promising chemical dimension. Zhu *et al.* introduced one n-type molecule, containing pyridine groups, into the perovskite precursor solution. The pyridinic nitrogen atoms coordinate with undercoordinated Pb^2+^ ions, while simultaneously forming hydrogen bonds with the –NH_2_ groups of FA^+^. This dual interaction not only saturates dangling bonds but also induces a surface transition of the perovskite from p-type to n-type *via* interfacial charge transfer, thereby suppressing nonradiative recombination by improving energy level alignment.^136^

At the perovskite surface, coordination and acid–base reactions occur spontaneously between surface ions and hydroxyl, amino, carboxyl groups on the transport layer surface, as well as residual solvents or additives, leading to the formation of a complex interfacial “reaction layer.” These interfacial chemical processes reshape the energy level alignment and, if left uncontrolled, result in reduced charge selectivity and enhanced nonradiative recombination. Active intervention of such interfacial reaction pathways through hydrogen-bond networks, coordination chemistry, or buffer layer design can replace thermodynamically spontaneous but detrimental side reactions with controllable and functionally optimized interface construction.

Suppressing the interfacial reactivity of organic cations at the source is a fundamental approach to block the formation of unfavorable intermediate phases.^138–141^ Lu *et al.* introduced the bis(2-oxo-3-oxazolidinyl) phosphinic chloride (BOP–Cl) into the perovskite precursor, whose design simultaneously targets both the perovskite bulk and the transport layer surface. In the bulk, the phosphoryl and two carbonyl groups of BOP–Cl form a six-membered hydrogen-bonded ring with FA^+^, which reduces the electrophilicity of ammonium protons and thus inhibits the acid–base reaction tendency between FA^+^ and hydroxyl groups on the SnO_2_ surface. Meanwhile, BOP–Cl forms a covalent protective layer on SnO_2_*via* a dechlorination reaction with nucleophilic oxygen atoms, replacing physical adsorption with chemical bonding and further stabilizing the transport layer interface.^142^ This strategy weakens the chemical potential of reactive species through supramolecular interactions in the bulk phase, thereby lowering the thermodynamic probability of interfacial side reactions. Beyond molecular additive strategies that modulate local chemical potentials, the deliberate incorporation of low-dimensional structural templates provides a combined physical and chemical pathway to suppress detrimental interfacial reactions. In a complementary approach, Sidhik *et al.* utilized a 2D perovskite with FA as the cage cation to serve as a lattice template for the low-temperature epitaxial growth of FAPbI_3_ (Fig. 4b) By pre-forming a stable two-dimensional interfacial scaffold, this method not only guides the oriented crystallization of the overlying 3D perovskite but also intrinsically mitigates the propensity for excessive reactivity of organic cations at the buried interface, thereby minimizing the formation of unfavorable intermediate phases from the earliest stages of film growth.^129^

From the perspective of interfacial ion distribution, the interplay of electric fields and chemical potential gradients drives halide and dopant ions to migrate and accumulate at interfaces. The resulting side reactions and defect state rearrangements are central mechanisms underlying interfacial chemical stability degradation. Chemically intervening in ion migration pathways through lattice engineering or coordination anchoring to suppress dopant escape has become a crucial strategy for stabilizing the interfacial chemical environment.

Lattice engineering to uniformly disperse halide ions offers an effective means to homogenize interfacial ion distribution from the source. As shown in Fig. 4c, Wang *et al.* proposed a “halide locking” strategy by introducing thioacetylacetamide hydrochloride (TAACl) into the perovskite precursor. Its strong binding with all cations and anions in mixed-halide perovskites effectively locks halide ions during the intermediate phase. The synergistic coordination between thioacetamide groups and Cl^−^ simultaneously targets Pb^2+^ and organic cations, achieving uniform halide distribution both vertically and horizontally.^143^ This kinetically suppresses the electric-field-driven halide accumulation at interfaces during device operation. Improving bulk halide distribution uniformity through lattice alloying similarly suppresses interfacial ion enrichment. Xu *et al.* employed a triple-halide alloying strategy (Cl, Br, and I), partially substituting I with Cl and tuning lattice parameters with Br, which significantly increased Cl solubility in the perovskite lattice (Fig. 4d).^144^ Since bulk defect sites often serve as low-barrier channels for ion migration, reducing bulk defects helps weaken the driving force for ion accumulation at interfaces.

On the HTL side, dopant ion migration at the interface also poses significant stability challenges.^127,145–147^ In conventional lithium bis(trifluoromethanesulfonyl)imide (Li-TFSI)/4-*tert*-butylpyridine (*t*BP) doping systems, Li^+^ migration and accumulation under thermal and electric fields are major causes of interfacial degradation. As shown in Fig. 4e, Kim *et al.* replaced volatile *t*BP with nonvolatile solid molecule 4-(*N*-carbazolyl)pyridine (4CP), which retains the Lewis basicity of the pyridinic nitrogen to coordinate Li^+^ while leveraging the carbazole fused aromatic backbone to enhance thermal stability and hole transport capability.^123^ This approach converts the Li^+^ coordination environment from a dynamically volatile to a static thermally stable state, suppressing the chemical potential-driven diffusion of Li^+^ toward the interface. Additionally, tuning dopant anion engineering is equally important. Shao *et al.* replaced linear TFSI^−^ with spherical large-radius Na-TFPB anions. The spherical TFPB^−^ anion, with its larger van der Waals radius and isotropic steric hindrance, significantly raises the migration barrier.^148^ These two dopant engineering strategies effectively suppress Li^+^ migration by stabilizing coordination and steric hindrance, whereas the 4CP-based approach is more scalable due to its compatibility with existing solution processing. Furthermore, in the more complex architecture of multi-junction tandem devices, suppressing defect-mediated phase segregation and ion redistribution is paramount for sustaining long-term interfacial integrity. As shown in Fig. 4f, the introduction of 3-ammonium propionic acid iodide (3AI) into the top subcell significantly reduces the formation of Schottky vacancies.^31^ This additive strategy elevates the phase-transition barrier of Br-rich perovskite compositions, thereby effectively retarding electric-field-driven halide separation and the associated interfacial degradation. Such findings extend the scope of interfacial stabilization from simple coordination chemistry to the modulation of intrinsic defect equilibria and phase stability in operational tandem devices.

In summary, the robust interfacial design lies not in the isolated optimization of a single coordination or doping pathway, but in the synergistic integration of these distinct chemical approaches. A unified framework, combining bulk lattice homogenization to suppress ion flux at the source with tailored, non-volatile coordination environments at the heterojunction, promises to decouple static passivation from dynamic stability.

#### 3.1.2 Halide-induced electrochemical corrosion and suppression in top interface and metal contact

At the metal/perovskite interface in PSCs, halide ions migrating to the interface undergo electrochemical reactions with reactive metal electrodes (such as Ag and Cu), forming non-conductive metal halides (*e.g.*, AgI and CuI). This leads to a sharp increase in contact resistance and ultimately causes interfacial failure. The core driving force of this chemical process lies in the redox potential difference between halides and metals. Halides act as oxidizing agents, converting metals into insulating metal halide layers that disrupt charge transport pathways. To interrupt this corrosive cycle from the perspective of interfacial chemistry, parallel strategies have been developed, including dense chemical barrier layers, inert electrode materials, and multilayer encapsulation barriers.

Using amorphous conductive nitrides as chemical barrier layers is an effective design that balances corrosion suppression and charge transport. Xiao *et al.* focused on Cu electrodes, introducing an amorphous zirconium nitride (amp-ZrN_*x*_) barrier layer between the perovskite and Cu. Electrochemical corrosion tests demonstrated that the protection efficiency of this barrier strongly depends on its degree of amorphization, as residual crystalline regions and their amorphous–crystalline interfaces often serve as fast ion permeation channels.^149^

From the intrinsic chemical inertness of electrode materials, substituting Cu with Cr as the first defense against halide corrosion is another approach to break the electrochemical cycle. Lanzetta *et al.* investigated the failure mechanism of Sn–Pb narrow-bandgap perovskites under reverse bias stress, revealing a complete chemical chain for Cu electrode degradation. Under reverse bias, iodide ions migrate to the interface and oxidize to I_2_, which reacts with I^−^ to form I_3_^−^. This leads to chemical corrosion of Cu, generating CuI. The resulting Cu^+^ ions migrate back to form conductive pathways, eventually causing hotspot-induced short circuits. Introducing a Cr layer significantly improves the device's thermal breakdown voltage by effectively blocking direct contact between I_2_ and Cu.^150^ This work experimentally traces the full reaction pathway of metal/perovskite interface degradation, providing a mechanistic blueprint that can be extended to diagnose and mitigate similar interfacial failures in other contact systems.

Extending these strategies to full device encapsulation, constructing multilayer inert barrier systems can isolate halides from metals at the source. Zhou *et al.* systematically compared the reactivity of various metal electrodes (Ag, Al, Cu, Au, and Bi) with halides, pointing out that reactive metals continuously consume halide decomposition products, disrupting the thermodynamic equilibrium of perovskite decomposition reactions and accelerating device degradation. They proposed replacing reactive metal electrodes with dense Bi films deposited by magnetron sputtering, combined with multilayer Al_2_O_3_/parylene thin-film barriers, to build a fully chemically inert system from electrode to encapsulation. Devices based on this multilayer barrier system retained 90% of their initial efficiency after 5200 hours of continuous illumination at 45 °C, and 93% after 1000 hours at 75 °C.^151^ This multilayer barrier strategy offers a generalizable design principle, combining a chemically inert metal (*e.g.*, sputtered Bi) with stepwise blocking layers, that can be adopted for stabilizing other perovskite devices against reactive metal-induced degradation. Another study introduced graded dielectric layers in perovskite/silicon tandem cells to mitigate the dielectric mismatch at the perovskite/C_60_ interface, thereby suppressing halide migration and degradation under reverse bias.^152^

In summary, the mitigation of electrochemical corrosion entails a trade-off between barrier interlayers and inert electrode foundations. Amorphous nitride films offer electronic tunability, yet their barrier integrity depends critically on microstructural uniformity. An inert electrode baseline, such as sputtered Bi with multilayer encapsulation, circumvents this constraint and directly eliminates the thermodynamic driving force for metal halide formation. These strategies therefore represent a more robust route to long-term interfacial stability.

### 3.2 Sn–Pb interface oxidation and the interconnecting layer (ICL) in multi-junction PSCs

Although multi-junction PSCs have achieved impressive efficiencies surpassing the Shockley–Queisser limit, their limited operational stability remains a major obstacle for commercialization. The stacked architecture inherently introduces complex interfacial issues. Recent progress in multi-junction PSCs is discussed, and effective approaches for realizing long-term operation are highlighted in Table 2.

**Table 2:** Summary of emerging strategies in multi-junction PSCs

Multi-junction PSCs	Methods	PCE (%)	Operational stability	Ref.
PVSK/silicon	HTL/PVSK	28.89%	*T*_67_, 300 h	146
PVSK/silicon	HTL/PVSK	33.86%	*T*_90_, 2000 h	153
PVSK/silicon	PVSK/ETL	31.6%	*T*_95_, 1000 h	154
PVSK/silicon	PVSK/ETL	33.89%	*T*_80_, 1200 h	155
PVSK/silicon	Additive (PVSK)	29.0%	*T*_86.6_, 306 h	135
PVSK/silicon	Additive (PVSK)	27.4%	*T*_93.6_, 450 h	147
PVSK/silicon	Additive (PVSK)	31.32%	*T*_95_, 1000 h	143
PVSK/silicon	Additive (PVSK)	30.78%	*T*_95_, 1000 h	156
PVSK/silicon	PVSK composition	27.13%	*T*_90_, 250 h	144
PVSK/silicon	Interlayer	29.30%	*T*_95_, 1000 h	157
PVSK/silicon	ICL	32.5%	*T*_90_, 870 h	158
PVSK/silicon	ICL	33.15%	*T*_91.7_, 1000 h	159
PVSK/silicon	ICL	29.2%	*T*_85_, 500 h	160
PVSK/silicon	ICL	32.32%	*T*_91_, 1000 h	161
PVSK/PVSK	Additive (PVSK)	27.90%	*T*_90_, 300 h	162
PVSK/PVSK	Additive (PVSK)	27.61%	*T*_90_, 1200 h	163
PVSK/PVSK	Additive (PVSK)	28.83%	*T*_90_, 1000 h	164
PVSK/PVSK	HTL/PVSK	29.63%	*T*_90_, 445 h	165
PVSK/PVSK	ICL	28.10%	*T*_90_, 500 h	166
PVSK/PVSK	ICL	24.40%	*T*_94_, 1000 h	167
PVSK/organic	PVSK/ETL	24.47%	*T*_90_, 500 h	25
PVSK/organic	ICL	24.07%	*T*_80_, 150 h	168
PVSK/organic	ICL	25.56%	—	169
PVSK/organic	ICL	23.2%	*T*_82.5_, 100 h	170
PVSK/organic	ICL	26.05%	*T*_80_, 650 h	171
PVSK/organic	ICL	25.34%	—	172
PVSK/organic	ICL	24.00%	*T*_80_, 130 h	24
PVSK/CIGS	HTL/PVSK	22.8%	*T*_77_, 400 h	173
PVSK/CIGS	ICL	26.10%	—	174
PVSK/CIGS	ICL	21.24%	*T*_80_, 120 min	175
PVSK/PVSK/Si	ICL	27.06%	*T*_95_, 407 h	176
PVSK/PVSK/Si	Additive (PVSK)	28.7%	*T*_80_, 260 h	31
PVSK/PVSK/PVSK	Additive (PVSK)	25.10%	*T*_80_, 200 h	41
PVSK/PVSK/PVSK	PVSK composition	24.33%	*T*_80_, 420 h	37

For multi-junction PSCs, particularly those incorporating narrow-bandgap Sn–Pb perovskites, such architectures introduces additional chemical complexity. Interfacial instability stems from the intrinsic oxidation propensity of Sn^2+^ in the narrow-bandgap sub-cell and the chemical interaction within the ICL, which acts as a “shared boundary” experiencing opposing ion fluxes from both sub-cells. In Section 3.2.1, we address Sn–Pb interface vulnerabilities, where Sn^2+^ oxidation accelerates structural degradation. This part reviews chemical strategies such as surface reconstruction, reductants/coordination agents, and crystallization control to suppress oxidation. In Section 3.2.2, we analyze the ICL as an active “chemical battlefield,” where bidirectional migration of halides, metal ions, and dopants leads to halide corrosion, Sn redox reactions, and metal diffusion-induced shunting. This subsection discusses design principles to manage these bidirectional fluxes and maintain ICL functionality and stability.

#### 3.2.1 Sn redox, defect chemistry and catalyzed decomposition in narrow-bandgap Sn–Pb perovskite interfaces

**Fig. 5:**
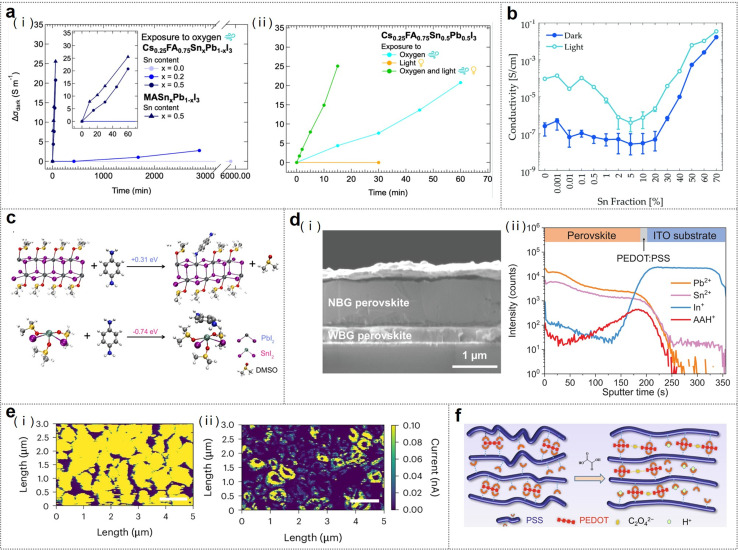
(a) Change in dark conductivity (Δ*σ*_dark_) of ASn_*x*_Pb_1−*x*_I_3_ films. (i) Δ*σ*_dark_ for varying A-cations and Sn fractions (*x* = 0.0, 0.2, 0.5), inset shows short-timescale response. (ii) Δ*σ*_dark_ for Sn_0.5_Pb_0.5_ under oxygen (blue), light (yellow), and both (green). Reproduced with permission from ref. 177. (b) Optoelectronic properties of (FA_0.83_Cs_0.17_)(Pb_1−*y*_Sn_*y*_)I_3_. Conductivity (four-point probe) in the dark and under ∼0.1 sun. Reproduced with permission from ref. 178, Copyright 2020, The Royal Society of Chemistry. (c) Simulated reaction energies between *p*-phenylenediamine (PPD) and PbI_2_·DMSO or SnI_2_·3DMSO solvate intermediates, highlighting selective Sn(ii) anchoring *via* cation–π interactions. Reproduced with permission from ref. 179, Copyright 2024, Springer Nature. (d) Aminoacetohydrazide hydrochloride (AAH) additive distribution in perovskite films. (i) Cross-sectional SEM of an all-perovskite tandem cell. (ii) TOF-SIMS profile showing AAH enrichment at the buried ITO/PEDOT:PSS interface. Reproduced with permission from ref. 180, Copyright 2024, American Association for the Advancement of Science. (e) Conductive atomic force microscopy (cAFM) images of mixed Sn–Pb perovskite films. (i) Control. (ii) Diamine, 1,2-diaminopropane (DAP)-treated film showing the homogenized surface Sn : Pb ratio. Scale bar, 1 µm. Reproduced with permission from ref. 164, Copyright 2024, Springer Nature. (f) Schematic of oxalic acid (OA)-induced self-assembly in PEDOT:PSS, forming ordered fibrous morphology *via* H^+^/PSS^−^ binding and C_2_O_4_^2−^/PEDOT^+^ bridging. Reproduced with permission from ref. 181, Copyright 2024, American Association for the Advancement of Science.

In narrow-bandgap tin–lead (Sn–Pb) perovskites, the oxidation of Sn^2+^ to Sn^4+^ represents a key chemical vulnerability, which is profoundly influenced by the interfacial chemical environment. Residual oxygen, moisture, and direct contact with oxide transport layers can catalyze this oxidation process, leading to carrier dynamics degradation and structural deterioration. Elucidating the oxidation mechanism induced synergistically by oxygen and illumination is a chemical prerequisite for designing effective suppression strategies. As shown in Fig. 5a, Nespoli *et al.* employed electrode-free microwave conductivity measurements to quantitatively analyze oxygen-induced doping kinetics in Cs_0.25_FA_0.75_Sn_0.5_Pb_0.5_I_3_ films, excluding interference from multilayer device interfaces. Their experiments demonstrated that oxygen acts as an electron acceptor at the perovskite surface, inducing Sn^2+^ oxidation to Sn^4+^, generating free holes and triggering metastable p-type doping, which requires approximately 2 days under a N_2_ atmosphere to revert to the initial dark conductivity, as inferred from the fitted data.^177^ However, the photoconductivity remained permanently degraded. From these observations, it can be inferred that in full devices where encapsulation limits further oxygen replenishment, the reversibility of doping is compromised at the level of charge carrier dynamics, and even transient oxygen exposure during fabrication can thus leave irreversible damage. This time-scale distinction provides a practical diagnostic framework for separately probing transient doping effects *versus* permanent structural degradation.

Based on this mechanism, synergistic suppression of oxidation pathways through interfacial coordination enhancement and reductive protection represents a direct chemical strategy to stabilize Sn–Pb perovskite interfaces. Bai *et al.* developed a dual system combining the polar phenylhydrazinium cation (PEH^+^) and the reductant 2-mercaptobenzimidazole (MBI). The design of PEH^+^ involves two chemical functions: its electronegative nitrogen atom strengthens electrostatic binding between –NH_3_^+^ and negatively charged defects, while strong coordination between PEH^+^ and Sn reinforces Sn–I bonds and increases the iodine ion migration barrier, thereby suppressing photoinduced I_2_ generation and subsequent Sn^2+^ oxidation at the source. The thiol group (–SH) of MBI forms hydrogen bonds with oxygen, enabling co-adsorption that significantly reduces charge transfer between O_2_ and Sn.^162^ These findings indicate that a single strategy is insufficient to address the dual oxidation channels induced by oxygen and photo-generated I_2_, so the stabilizing interfacial Sn^2+^ requires simultaneous chemical approaches of “strengthening intrinsic bonding” and “exogenous reductive protection.”

Besides, the higher intrinsic defect density and more active ion migration may lead to facile formation and bulk propagation of reactive interfacial layers, constituting a core chemical bottleneck limiting device performance and stability. From quantitative defect chemistry diagnosis to crystallization kinetics modulation *via* coordination, and chemical reconstruction of surface composition, researchers have established multilevel strategies spanning mechanistic understanding to interfacial intervention.

Revealing the non-monotonic correlation between Sn content and defect density is a chemical basis for understanding Sn–Pb perovskite interface fragility. Klug *et al.* systematically investigated the optoelectronic properties of (FA_0.83_Cs_0.17_)(Pb_1−*y*_Sn_*y*_)I_3_ across the full compositional range, identifying a critical “defect window.” Electrical transport measurements reveal a non-monotonic evolution of conductivity with Sn incorporation: dark conductivity initially decreases from ∼10^−7^ S cm^−1^ for the pure-Pb film to a minimum of ∼10^−8^ S cm^−1^ at 5% Sn, then increases sharply beyond 20% Sn, exceeding 10^−2^ S cm^−1^ in Sn-rich compositions. This indicated that low-level Sn incorporation may act as a compensating defect or scattering center, whereas higher Sn fractions promote a substantial increase in mobile charge carriers (Fig. 5b).^178^ This work establishes the non-monotonic relationship between Sn content and defect activity, providing clear guidance for compositional selection in narrow-bandgap devices. Additionally, Ambrosio *et al.* conducted in-depth calculations on MAPb_0.5_Sn_0.5_I_3_ surfaces, revealing thermodynamic and kinetic competition between surface Sn(iv) formation and iodine-related defects such as triiodide (I_3_^−^). Under iodine-rich conditions, Sn vacancies become the most stable surface defects, whereas under Sn-rich conditions, the formation energy of hole-trapping defects significantly increases, greatly suppressing defect activity.^182^ This finding explains the beneficial effect of Sn excess powder observed experimentally.

From the perspective of coordination chemistry, modulating crystallization kinetics is an effective approach to achieve uniform Sn–Pb alloying. Zhang *et al.* identified a deep chemical origin limiting Sn–Pb perovskite film quality: the coordination energy of Sn(ii) with DMSO is much lower than that of Pb(ii), causing Sn-based components to crystallize faster than Pb-based ones, leading to asynchronous crystallization and compositional inhomogeneity. Based on this insight, they introduced *p*-phenylenediamine (PPD) as a non-covalent binder, selectively anchoring coordinatively unsaturated Sn(ii) complexes *via* cation–π interactions. The simulated reaction energetics are illustrated in Fig. 5c.^179^ This finding attributes asynchronous Sn–Pb crystallization to the coordination chemistry characteristics determined by Sn(ii) lone pairs and achieves kinetic synchronization of Sn–Pb alloying.

Complementary to bulk crystallization control, chemical passivation of surface Sn enrichment is also an effective strategy for interface stabilization. Gao *et al.* screened aminoacetohydrazide hydrochloride (AAH) as an additive, which delays crystallization through comprehensive supramolecular interactions with PbI_2_, SnI_2_, FAI, and DMF solvent, and spontaneously enriches itself at the buried interface during crystallization due to its high polarity, with confirmed Lewis acid–base adduct formation. The cross-sectional morphology and interfacial enrichment of AAH are shown in Fig. 5d.^180^ Li *et al.* observed significant Sn enrichment gradients (Sn : Pb atomic ratio ∼4 : 1) on mixed Sn–Pb perovskite film surfaces, exacerbating surface oxidation. They employed diamine ligands with preferential chelation to Sn, adjusting the surface Sn : Pb ratio to approximately 1 : 1, achieving more uniform chelate distribution, stabilizing the Sn^4+^ content around 5% for over 12 hours. The corresponding conductive atomic force microscopy (cAFM) images are provided in Fig. 5e.^164^ Both studies provide a transferable design principle: selective coordination and spatial arrangement of functional molecules at the interface can upgrade defect management from passive tolerance to active regulation.

In narrow-bandgap Sn–Pb perovskites, the accumulation of Sn^2+^ oxidation products (Sn^4+^ species and SnO_*x*_) and related halides at interfaces forms a complex chemical microenvironment. This alters interfacial energy level alignment and ion transport barriers, causing long-term electrical drift that critically limits device stability.

Introducing polar Lewis base additives into perovskite precursors to regulate crystallization kinetics *via* supramolecular interactions and suppress Sn^2+^ oxidation is an effective chemical approach to block side product formation at the source. Chen *et al.* addressed the poor buried interface contact and non-uniform Sn–Pb perovskite crystallization caused by the hydrophobicity of the PTAA HTL by introducing the sulfur-urea-based Lewis base additive 4-bromophenylthiourea (BPSU). BPSU forms strong hydrogen bonds with FAI and coordinates more strongly with SnI_2_ than with PbI_2_, preferentially delaying Sn-based component crystallization and effectively balancing asynchronous Sn–Pb crystallization kinetics.^163^ It realizes selective coordination of Sn components by additives.

Beyond additive strategies, chemical modification of the HTL to optimize interfacial energy levels and morphology can indirectly suppress side product-induced disturbance of the interfacial electronic structure. Zhu *et al.* treated poly (3,4-ethylenedioxythiophene)-poly (styrenesulfonate) (PEDOT:PSS) HTLs with oxalic acid (OA), whose high ionization constant generates protons and oxalate ions. Through electrostatic binding between H^+^ and PSS^−^ and bridging interactions between C_2_O_4_^2−^ and PEDOT^+^, an ordered fibrous phase-separated morphology is formed. A schematic of this self-assembly process is depicted in Fig. 5f.^181^ This chemical modification of the transport layer exemplifies indirect regulation of side product effects from the perspective of interfacial physicochemical environment. Introducing side-chain engineering into a self-assembled monolayer (SAM) design enables molecular-scale optimization of both interfacial charge extraction and perovskite crystallization quality. Zhu *et al.* synthesized three SAM hole-selective layers based on a donor–acceptor molecular backbone with benzothiadiazole units functionalized by oligomeric ether side chains of varying lengths. Density functional theory (DFT) calculations show that oligomeric ether side chains coordinate more strongly with Sn^2+^ than Pb^2+^, preferentially binding Sn components to regulate crystallization kinetics of Sn-containing perovskites.^183^ This work establishes a transferable design paradigm: the oligomeric ether side-chain length serves as a tunable molecular parameter, enabling self-assembled monolayers to synergistically achieve hole extraction, crystallization regulation, and defect passivation across other perovskite interfaces.

In narrow-bandgap Sn–Pb perovskites, stabilizing interfaces requires simultaneous management of Sn^2+^ oxidation, asynchronous crystallization, and surface Sn enrichment. Reductive additives and selective coordination agents mitigate degradation pathways, yet their effectiveness hinges on synchronizing Sn and Pb crystallization kinetics. Integrating crystallization-modulating Lewis bases with chemically tailored transport layers thus offers a coherent route toward converting the interface into an actively stabilized chemical environment. A recent review by Gao *et al.* has systematically surveyed interface engineering strategies for Pb–Sn PSCs and all-perovskite tandem solar cells, with a particular emphasis on grain boundary, buried interface, and top surface modification.^184^ It offers a device-level engineering perspective that complements our focus on the interfacial chemical reactions, such as Sn^2+^ oxidation pathways and crystallization kinetics, that underpin interface degradation and stabilization.

#### 3.2.2 Bidirectional chemical fluxes and degradation in the ICL

In tandem solar cells, the ICL serves as the “shared boundary” between the top and bottom subcells, enduring bidirectional migration and accumulation of halide ions, metal species, and dopants from both sides. This chemical process not only disturbs interfacial dipole distributions and band alignment but may also trigger electrochemical side reactions and long-term electrical drift, posing a central interfacial chemical challenge that limits tandem device stability.

**Fig. 6:**
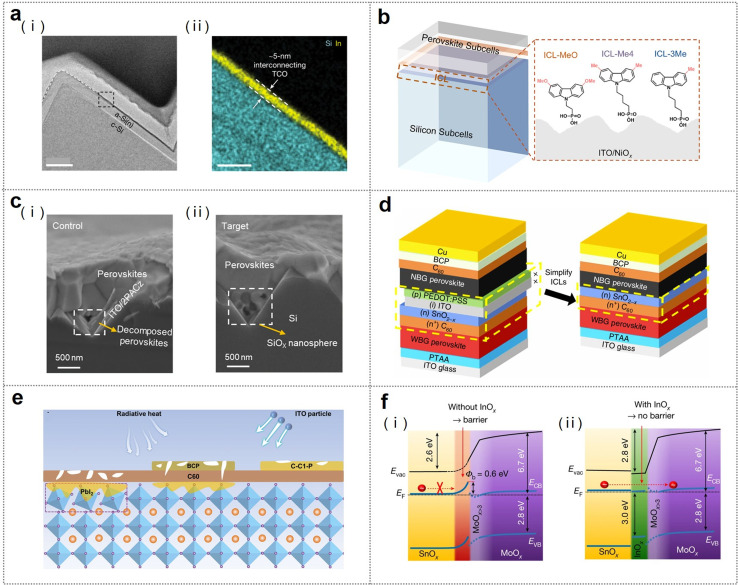
(a) Cross-sectional TEM image (i) of the ITO layer on pyramidally textured c-Si bottom cells (scale bar, 100 nm), and the corresponding EDS mapping (ii) of the selected area of an ITO sample (scale bar, 20 nm). Reproduced with permission from ref. 158, Copyright 2023, Springer Nature. (b) Schematic illustration of perovskite/silicon tandem PSCs, highlighting the ICL structure at the perovskite/ICL interface. Reproduced with permission from ref. 161, Copyright 2025, Elsevier Inc. (c) Cross-sectional SEM images of perovskites on submicron-textured silicon substrates (control, (i)), and SiO_*x*_-modified (target, (ii)) substrates after heat treatments. Reproduced with permission from ref. 159, Copyright 2025, Nature Springer. (d) Schematic diagram of perovskite/perovskite tandem PSCs featuring a typical structured ICL (C_60_/SnO_2−*x*_/ITO/PEDOT:PSS) and a simplified ICL (C_60_/SnO_2−*x*_) for monolithic all-perovskite tandem PSCs. The arrow indicates the decrease in the number of layers in the proposed ICL structure. Reproduced with permission from ref. 167, Copyright 2020, Springer Nature. (e) Schematic illustration of the protection mechanism preventing a perovskite from damage during the ITO sputtering process. Reproduced with permission from ref. 168, Copyright 2023 Wiley-VCH GmbH. (f) Energy level alignment determined (i) without and (ii) with InO_*x*_ layer. Reproduced with permission from ref. 24, Copyright 2022, Springer Nature.

Controlling the interfacial electric field and ion flux by tuning the thickness of TCO and dopant distribution is an effective electrochemical–chemical strategy to stabilize the ICL. As shown in Fig. 6a, Aydin *et al.* focused on the thickness effect of indium zinc oxide (IZO) in the ICL and revealed a profound correlation between TCO resistance and interfacial chemical environment. Their experiments showed that reducing the IZO thickness from 20 nm to 5 nm increases the sheet resistance by two orders of magnitude. This change brings dual interfacial optimization. First, the high lateral resistance effectively limits current shunting caused by submicron perovskite defects. Second, the 5 nm IZO layer induces a stronger interfacial electric field and upward band bending at the perovskite/(2-(9*H*-carbazol-9-yl)ethyl)phosphonic acid (2PACz) interface, suggesting suppressed carrier recombination and enhanced hole collection efficiency.^158^ This strategy clarifies that the chemical dipole density and uniformity at the TCO/SAM interface directly regulate the interfacial potential distribution, thereby influencing the migration and redistribution dynamics of dopant ions such as Li^+^ within the ICL region.

Blocking direct contact between halides and TCO *via* interfacial molecular engineering can also suppress corrosion at its source. Wang *et al.* addressed halide migration and interfacial corrosion caused by “wet spots” in perovskite/TOPCon silicon tandem devices by modifying the metal oxide surface with an asymmetric phosphonate molecule, (4-(3-methyl-9*H*-carbazol-9-yl)butyl)phosphonic acid (3-Me-4PACz), which possesses a large dipole moment. The asymmetric methyl substitution enhances dipole–dipole interactions with polar solvents, significantly improving interfacial wettability (Fig. 6b).^161^ This molecular design demonstrates that large dipole moments not only optimize interfacial energy level alignment but also inhibit dewetting during the wet film stage by increasing surface adhesion energy, thus eliminating preferential pathways for halide migration. Tandem devices based on this strategy achieved a certified efficiency of 32.32% and an open-circuit voltage of 2.023 V. As shown in Fig. 6c, in a complementary approach focusing on the bottom silicon subcell, Zhang *et al.* introduced a localized submicron silica nanosphere (SiO_*x*_) layer to improve uniform perovskite deposition on textured silicon surfaces, thereby promoting a stable perovskite/silicon interface and reducing interfacial recombination losses in tandem PSCs.^159^

Within the ICL of perovskite tandem solar cells, halides, Sn-related species, and metal dopants from the top and bottom subcells converge at the shared boundary, leading to competing interfacial chemical reactions. Halide-induced corrosion of TCO, redox cycling of Sn species, and metal diffusion causing short-circuit risks intertwine to form the chemical root of ICL interfacial failure.

An effective approach to actively manage interfacial chemical reactions is to construct a simplified ICL based on the bipolar carrier transport properties of SnO_2−*x*_, by controlling the stoichiometry and electronic states of the oxide layer. Yu *et al.* replaced the conventional complex multilayer ICL structure with low-temperature atomic layer deposited amorphous SnO_2−*x*_, retaining only a C_60_/SnO_1.76_ bilayer to achieve efficient carrier recombination. Perovskite-only tandem devices based on this ICL reached a stabilized efficiency of 24.4%, retaining 94% of the initial performance after 1000 hours of illumination (Fig. 6d).^167^ This strategy indicates that Sn^2+^ can act as an electron donor participating in interfacial charge balance.

From the perspective of ICL structural design, replacing traditional TCO-based composite layers with tunnel junctions effectively avoids the dual risks of halide-oxide reactions and metal diffusion. Zheng *et al.* innovatively integrated a polycrystalline silicon tunnel junction into perovskite/TOPCon tandem devices. By suppressing dopant interdiffusion and compensation, the tunnel junction forms a sharp B/P doping boundary at the poly-Si(p)/poly-Si(n) interface, ensuring efficient band-to-band tunneling.^160^

Compared to multilayer ICL strategies, simplifying the ICL structure itself is an effective way to reduce interfacial reaction sites. Xiao *et al.* employed thermally evaporated YbO_*x*_/Au/MoO_*x*_ as a simplified ICL to replace the conventional SnO_*x*_/IZO/PEDOT:PSS multilayer structure in perovskite/organic tandem cells. This approach replaces atomic layer deposition (ALD) and sputtering processes with thermal evaporation, reducing chemical damage to the underlying perovskite by high-energy particles. Using a single YbO_*x*_ layer instead of complex oxide stacks reduces the number of halide-corrodible oxide interfaces at the source.^170^ Alternatively, the ICLs can be polymerized to form an insoluble network that shields the underlying organic layer from damage by high-energy sputtering species, thereby combining physical protection with efficient charge transport. As shown in Fig. 6e, the ICLs can be polymerized to form an insoluble network that shields the underlying organic layer from damage by high-energy sputtering species, thereby combining physical protection with efficient charge transport.^168^

Interfacial failure in ICLs follows a clear chemical causality chain. The chemical evolution of the interfacial reaction layer leads to increased ionic conductivity and intensified migration, which in turn cause reduced interfacial charge selectivity and enhanced nonradiative recombination, ultimately resulting in electrical degradation of the open-circuit voltage and fill factor. Starting from defect chemistry in the interfacial oxide layer, it is revealed that oxygen vacancy-induced metallization transition is a key lever for tuning ICL electrical behavior. Brinkmann *et al.* addressed the 0.6 eV Schottky barrier at the SnO_*x*_/MoO_*x*_ interface in perovskite/organic tandem cells by introducing an ultrathin (∼1.5 nm) ALD InO_*x*_ layer to form a SnO_*x*_/InO_*x*_/MoO_*x*_ trilayer with ohmic contact (Fig. 6f).^24^ By precisely controlling the InO_*x*_ thickness at the nucleation critical thickness, this work achieves a balanced optimization of defect chemistry and electrical function.

At metal nanoparticle interfaces, the coverage and morphology evolution of Au nanoparticles directly correlate with the thickness of the interfacial reaction layer and ionic conductivity. Zheng *et al.* systematically studied the deposition behavior of ultrathin Au on ALD-SnO_2_ surfaces and its interfacial electrical consequences. Transmission electron microscopy (TEM) characterization revealed that Au forms discrete nanoparticles rather than continuous films, with nanoparticle coverage increasing over deposition time.^176^ This approach establishes a generalizable coverage-threshold principle for metal/oxide interfaces, guiding the design of transparent electrodes by balancing charge selectivity and metal diffusion risks.

From the perspective of barrier layer design, maintaining the intrinsic chemical stability of the interfacial chemical structure can block the vicious cycle of rising ionic conductivity. The previously mentioned work by Xiao *et al.* on chemical corrosion and ion penetration at the Cu/perovskite interface, which introduced an amorphous ZrN_*x*_ barrier layer, also validates the effectiveness of blocking failure chains from the chemical source.^149^

As the central electrical and chemical junction between subcells, the ICL critically governs tandem device stability. Long-term robustness ultimately reflects a fundamental compromise between increased device complexity and interfacial chemical resilience. Simplified ICL architectures reduce the density of reactive sites and suppress degradation pathways originating from both subcells, offering a more reliable route toward durable tandem operation. However, this simplification is not free of trade-offs. Overly reduced interlayer thickness or the use of less conductive materials may increase parasitic absorption or compromise carrier recombination efficiency, potentially raising series resistance and lowering fill factor. Therefore, achieving an optimal balance between chemical simplicity and electrical functionality requires careful tuning of layer thickness, conductivity, and optical transparency to ensure that reduced interfacial reactivity does not come at the expense of charge extraction and light management.

## 4 Chemistry-to-function design rules for interfacial stabilization

Building on the degradation mechanisms analyzed in the previous section, this section distills design rules that translate chemical insights into stable interfacial functions. Coordination passivation, which modulates defect-state thermodynamics and kinetics at the molecular level (Section 4.1), and barrier/interphase engineering, which controls the macroscopic fluxes of halides, metals, and dopants (Section 4.2) are emphasized here. Together, they move beyond mere “hole filling” toward chemically grounded, function-oriented interface stabilization.

### 4.1 Coordination passivation: modulating defect states and interfacial chemistry

In studies of perovskite interface stability, coordination passivation, a core strategy of Lewis acid–base chemical engineering, has evolved from simple “defect filling” to the deliberate design of precise chemical control over defect thermodynamics and kinetics. For Lewis acid–base sites such as undercoordinated Pb^2+^, halide vacancies, and A-site vacancies, designing an effective passivating molecule requires the simultaneous optimization of multiple interdependent criteria: binding energetics, site selectivity, molecular orientation, and operational chemical inertness.

**Fig. 7:**
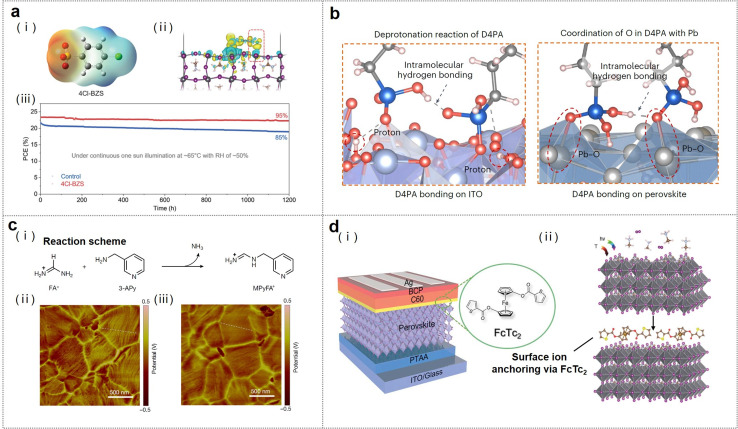
(a) Planar-oriented 4-chlorobenzenesulfonate (4Cl-BZS) ligand for dual-site defect passivation in perovskites. (i) Electrostatic potential of the 4Cl-BZS molecule; (ii) coordination with Pb^2+^ defect sites *via* a planar geometry, with reduced energetic losses and improved charge transport; (iii) maximum power point tracking of control and 4Cl-BZS-based PSCs. Reproduced with permission from ref. 106, Copyright 2024, American Association for the Advancement of Science. (b) Enlarged view of the phosphonic acid moiety in the D4PA molecule bonded to a perovskite, showing the energy variations among rotational conformations and the calculated optimal structure. Reproduced with permission from ref. 111, Copyright 2025, Springer Nature. (c) Surface n-type doping induced by 3-(aminomethyl)pyridine (3-APy): (i) reaction scheme of 3-APy with formamidinium (FA^+^) ions on the perovskite surface; (ii and iii) Kelvin probe force microscopy images of the perovskite film with and without 3-APy surface treatment, respectively. Reproduced with permission from ref. 107, Copyright 2022, Springer Nature. (d) Accelerated electron transfer *via* ferrocenyl-bis-thiophene-2-carboxylate (FcTc_2_) modification: (i) inverted PSC structure using FcTc_2_ as the interface functional layer and (ii) schematic illustration of surface ions by FcTc_2_. Note: *T*, temperature; *hν*, photon energy. Reproduced with permission from ref. 185, Copyright 2022, American Association for the Advancement of Science.

Starting from the adsorption configuration and binding energy of passivating molecules, controlling coordination orientation is a crucial chemical dimension to optimize interfacial charge transport. As shown in Fig. 7a, Chen *et al.* systematically compared the adsorption behavior of aromatic sulfonate ligands on perovskite surfaces, finding that the introduction of a chlorine substituent in 4-chlorobenzenesulfonate (4Cl-BZS) causes the molecule to preferentially adopt a configuration parallel to the surface, unlike benzenesulfonate and 4-methylbenzenesulfonate which tend to adsorb vertically. DFT calculations revealed that this configurational change arises from additional coordination between the Cl atom and surface Pb^2+^, enabling 4Cl-BZS to passivate Pb^2+^ sites at two points simultaneously.^106^ Importantly, this work emphasizes that the stability of passivating molecule configurations must be validated under operating conditions; the advantage of parallel adsorption must be maintained under illumination, bias, and thermal stress, imposing stringent requirements on the reversible adsorption/desorption kinetics of passivators.

Suppressing aggregation and migration of passivators through molecular design is another key to maintaining interfacial chemical uniformity. Zheng *et al.* studied phosphonate molecules substituted with multiple methyl or methoxy groups (*e.g.*, 4-methyl-4-phenyl-1,3-diazole (Me-4PACz), 4-(3,6-dimethoxy-9*H*-carbazol-9-yl)butyl]phosphonic acid (MeO-4PACz)), finding that larger molecular size and enhanced lateral van der Waals interactions promote the formation of highly grafted, ordered SAMs on ITO surfaces. This improved the fill factor from 60–65% with 2PACz to approximately 80% with Me-4PACz.^186^ Gao *et al.* further achieved dual anchoring coordination at the interface using a C–C bonded bis-carbazole phosphonate (D4PA), whose strong binding with perovskite forms early during grain growth and remains stable. Dynamic light scattering confirmed that intramolecular steric hindrance effectively suppresses solution-phase aggregation of the passivator (Fig. 7b).^111^ Together, these studies offer a transferable design principle that the interfacial stability of passivators is governed by intermolecular forces and conformational rigidity, where enhanced steric hindrance and anchoring synergy can be broadly adopted to suppress molecular migration and performance drift under operation.

Extending the coordination network from single molecules to crosslinked supramolecular structures can further enhance the thermal stability and mechanical robustness of interfacial passivation. Wang *et al.* designed a fluorinated, thermally crosslinkable SAM molecule, whose carbazole core is terminated on both sides with 4-vinylphenyl groups that undergo *in situ* crosslinking at mild temperatures to form a continuous polymer network.^187^ This crosslinked network not only strengthens the SAM layer's resistance to solvent erosion but also locks molecular configurations covalently, preventing rearrangement and migration of passivators under thermal stress.

Unlike pre-formed passivation, *in situ* defect chemical reconstruction induced by reactive molecules at the interface opens new pathways to regulate defect charge states and built-in electric fields. As shown in Fig. 7c, Jiang *et al.* employed 3-aminomethylpyridine (3-APy) as a reactive passivator, which undergoes a condensation reaction between its amine group and surface FA^+^ to form *N*-(3-methylpyridine)formamidinium (MPyFA^+^) cations while releasing NH_3_. Positively charged iodine vacancies act as shallow donors electrostatically with the pyridine ring of MPyFA^+^, jointly establishing an interfacial built-in electric field. Devices based on this strategy retained 87% of their initial efficiency after 2400 hours of maximum power point tracking at 55 °C.^107^ Li *et al.* also used ferrocene-based bis-thiophene carboxylate (FcTc_2_) as an interfacial functional molecule, with DFT simulations showing stable Pb–O coordination between its oxygen atoms and Pb^2+^. Another important contribution of this work is the chemical tracking of passivation stability: peak force infrared (PFIR) microscopy mapping revealed that after 1000 hours of photothermal aging, the MA^+^ cation signal intensity and distribution remain intact in FcTc_2_-treated samples, while significant attenuation and broadening occur in controls. Devices based on this strategy achieved 25.0% efficiency and passed the silicon cell international standard in damp heat testing at 85 °C/85% RH (Fig. 7d).^185^

In summary, the progression from static defect filling to deliberate interfacial design establishes that molecular architecture directly governs long-term stability under operational stress. Rational control of adsorption geometry, steric hindrance, and crosslinked network formation transforms the passivating layer into a structurally resilient and electronically favorable interphase. Such designed interfaces retain configurational integrity under illumination and thermal cycling, thereby isolating defect healing from performance drift and providing a robust chemical basis for durable devices.

### 4.2 Controlling chemical fluxes for barrier and interphase design

In PSCs, the design of dense blocking layers should transcend mere physical densification. Rather, it must be engineered around three explicit chemical design criteria: (i) suppressing the diffusion coefficients of halides and metals, (ii) rendering the interfacial reaction free energy thermodynamically unfavorable for corrosion product formation, and (iii) precluding the formation of ion-conductive pathways under operational electric fields. Exploiting the intrinsic reactivity of metal oxides to guide interfacial reactions thus becomes the foundational principle for the rational selection and design of barrier materials.

**Fig. 8:**
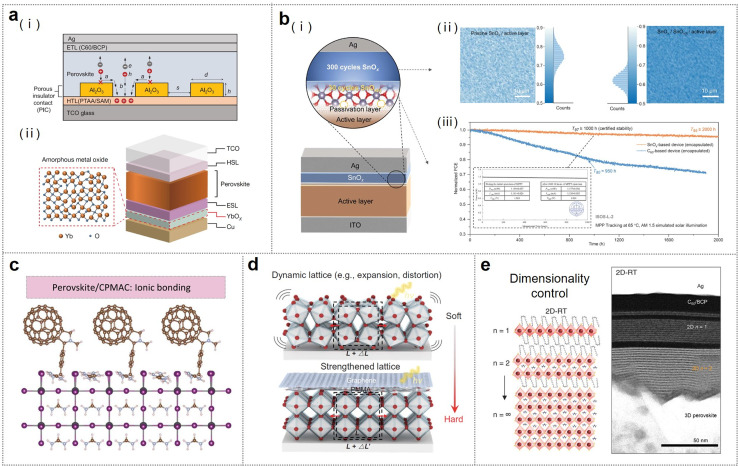
(a) Device architectures of (i) Al_2_O_3_-based and (ii) YbO_*x*_-based PSCs. Reproduced with permission from ref. 117, Copyright 2023, American Association for the Advancement of Science, and ref. 108, Copyright 2024, Springer Nature, respectively. (b) (i) Schematic illustration of the atomic layer deposited (ALD)-SnO_*x*_ based PSC device. (ii) PL mapping of the pristine SnO_*x*_/active layer and the SnO_*x*_/SnO_*x*(i)_/active layer. (iii) Operational stability of the ALD-SnO_*x*_ based PSC at 65 °C. Reproduced with permission from ref. 109, Copyright 2024, American Association for the Advancement of Science. (c) Interactions between CPMAC molecules and the perovskite lattice through ionic bonding. Reproduced with permission from ref. 110, Copyright 2025, American Association for the Advancement of Science. (d) Structural variations of the perovskite lattice induced by light irradiation. Black dashed rectangles and red arrows denote lattice expansion measured by the change in length (*L*). Reproduced with permission from ref. 104, Copyright 2025, American Association for the Advancement of Science. (e) Dimensionality control of 2D perovskite passivation with different n layers under a thermal annealing at 100 °C and room temperature process, along with the corresponding cross-sectional high-resolution scanning transmission electron microscopy (HR-STEM) image. Reproduced with permission from ref. 130, Copyright 2022, American Association for the Advancement of Science.

Regulating interfacial reactions by leveraging the intrinsic properties of metal oxides forms the basis for selecting blocking layer materials. Yang *et al.* systematically investigated the interfacial reactivity of various metal oxides with perovskites and found that the acid–base strength of the material directly determines its chemical reaction tendency with the perovskite. Mass spectrometry analysis showed that ALD AlO_*x*_ layers, due to their acidic nature, significantly suppress the release of organic decomposition products at the interface compared to NiO_*x*_ substrates.^115^ The study not only provides an acid–base criterion for screening blocking layer materials but also reveals the unique role of fixed negative charges in AlO_*x*_. Chen *et al.* used thermally evaporated YbO_*x*_ as a model buffer layer and tracked the oxidation kinetics from Yb^0^ to Yb^3+^*via in situ* X-ray photoelectron spectroscopy (XPS). Ultraviolet photoelectron spectroscopy (UPS) depth profiling revealed that inserting YbO_*x*_ reduces the contact interface work function. Carrier transport measurements confirmed that its conduction mechanism follows a thermally activated hopping model through localized states.^108^ In addition, porous insulating and metal oxide buffer layers (Fig. 8a), such as aluminum, silicon, zirconium, and ytterbium oxides, have been employed, playing an important role in balancing carrier recombination and charge transport as functional barriers.^108,117^ These strategies achieve a chemical separation of ion blocking and charge transport.

Blocking direct chemical contact between oxides and perovskites *via* buffer layers is an effective means to suppress detrimental interfacial reactions and band misalignment. Gao *et al.* introduced ALD-SnO_*x*_ as an inorganic ETL and incorporated an aliphatic amine-functionalized perylene diimide (PDINN) as a buffer layer. When oxygen vacancies exist in SnO_*x*_, the spatial charge density associated with these vacancies activates carrier distribution in the conjugated aromatic core of PDINN, enhancing conduction band minimum band dispersion and alleviating the “cliff-like” band misalignment caused by direct contact. XPS analysis confirmed that PDINN effectively prevents direct reactions between H_2_O and the perovskite (Fig. 8b).^109^ The ingenuity of this design lies in transforming oxygen vacancies from potential interfacial instability factors into chemical switches that activate electronic interaction in the buffer layer. As shown in Fig. 8c, a fullerene-derived ionic salt functions as an electron shuttle to create ideal interfacial and mechanical perovskite/ETL contacts, enabling module-level PSCs to retain over 91% of their initial efficiency after 2200 hours of operation at 55 °C.^110^ Moreover, a polymer monolithic graphene interlayer mechanically reinforces the perovskite films, increases their modulus and hardness, reduces tensile strain, and minimizes structural degradation from light-induced lattice expansion (Fig. 8d).^104^

Similarly, *in situ* induction of a two-dimensional (2D) phase transformation on the perovskite surface can construct functionalized interfaces. As shown in Fig. 8e, Azmi *et al.* performed post-treatment of 3D perovskite surfaces with oleylammonium iodide (OLAI). Through coordination between amine ligands and Pb^2+^ ions, combined with I^−^ ion exchange, a Ruddlesden–Popper type 2D perovskite layer is formed. This 2D interlayer finely tunes the interfacial energy levels, enhancing n-type characteristics and significantly improving the interfacial charge transfer efficiency. Additionally, the hydrophobicity of long alkyl chains provides a physical barrier that delays water and oxygen ingress.^130^ Further optimization of preparation conditions to enrich high-n 2D phases is key to fully exploiting the potential of such energy level engineering.

In conclusion, effective barrier interphases must achieve chemical control of ion flux and interfacial reactivity rather than mere physical densification. From a scalability perspective, approaches that exploit intrinsic material properties or spontaneously forming interlayers offer clear manufacturing advantages over complex multilayers requiring precise thickness control. Solution based strategies that generate functional interphases through chemical reactions align naturally with large-scale manufacturing. Prioritizing such chemically driven and self-assembled interphases therefore provides a pragmatic route toward scalable and durable perovskite devices.

## 5 Summary and outlook

This review has systematically examined the interfacial degradation chemistry in both single-junction and multi-junction PSCs, covering aspects such as molecular coordination, SAMs, barrier layers, and ICLs. Nevertheless, these numerous strategies prompt a critical question: which of them are truly essential for advancing toward commercial application?

For single-junction PSCs, the primary threats are the buried interface with the transport layer and the corrosion of the metal electrode. Here, the most practical solutions involve simple yet chemically robust modifications. For instance, multifunctional SAMs offer a straightforward means to passivate defects and improve adhesion without introducing complex processing steps. Similarly, replacing reactive silver or copper electrodes with more inert materials, or inserting a thin, dense barrier layer, can effectively disrupt the cycle of halide-induced corrosion. These approaches directly address the main failure points of single-junction devices and provide a clear route toward scalable manufacturing. In multi-junction architectures, the challenge becomes more complex. For tandem cells that employ narrow-bandgap tin–lead perovskites, the central concerns shift to tin oxidation and the chemical instability of the ICL. In this context, a key direction is structural simplification. Replacing the conventional multilayer interconnecting stack with a simpler tunnel junction or a minimal bilayer recombination contact directly reduces the number of vulnerable interfaces and eliminates many sites where detrimental interfacial reactions may occur. These strategies are no longer limited to passive defect filling; instead, they aim to actively regulate ion migration and interfacial chemical reactions, thereby mitigating long-term device degradation.

Despite considerable progress, our understanding of how increasing device complexity reshapes interfacial chemical behavior remains incomplete. The transition from single-junction to multi-junction tandem cells is not merely an additive superposition of degradation pathways; rather, it gives rise to an interaction network that integrates chemical, mechanical, and electrical processes. It is therefore imperative to employ advanced structural, optical, and electrical *operando* characterization techniques to elucidate the dynamic mechanisms of interfacial degradation and clarify the degradation pathways, thereby establishing a theoretical foundation for the rational design of interface-stable devices. Building upon this understanding, molecular design of interfacial materials and optimization of deposition processes constitute the central strategies for enhancing interface stability. However, conventional trial-and-error approaches are increasingly inadequate to address the growing complexity of materials design. Future efforts should integrate density functional theory, molecular dynamics simulations, high-throughput screening, and machine learning with *operando* characterization techniques to develop standardized materials databases and design guidelines, facilitating a paradigm shift from experience-driven to data-driven research. As an encouraging sign, machine learning has already been employed to screen interfacial passivators and predict their stabilization efficacy in perovskite systems, with early successes in identifying effective buried-interface modifiers and candidate passivants. Furthermore, integrating machine learning with automated fabrication has enabled the efficient discovery of high-performance passivation molecules with greatly improved reproducibility.^188^ Nevertheless, challenges remain in building standardized interfacial databases and achieving robust model transferability across diverse perovskite compositions, which must be overcome to fully realize this data-driven paradigm. Furthermore, the scaling-up of device fabrication introduces additional challenges to interface engineering. As device sizes increase, issues such as non-uniform coverage of interfacial materials, localized strain accumulation, and defect aggregation become increasingly pronounced. For example, while techniques such as solution-based SAM formation perform well on small laboratory samples, they often fail to produce uniform, defect-lean films over large-area modules. For these critical interfacial components, transitioning to vapor-phase deposition will likely be essential for achieving the reliability and yield demanded by industrial production. Consequently, future work should focus on optimizing scalable deposition techniques such as slot-die coating, blade coating and vapor-phase deposition, developing in-line process monitoring and quality control methodologies, and designing interfacial material systems with high tolerance to process variations, thereby enabling the effective translation of laboratory-scale achievements into industrial manufacturing.

In summary, the future of perovskite photovoltaic technology hinges on addressing the core challenges in interface science. Future research urgently requires a deepened understanding of interfacial degradation mechanisms, the adoption of artificial intelligence-driven materials design paradigms, the implementation of multi-interface synergistic optimization strategies, the innovation of interfacial deposition techniques, and breakthroughs in overcoming the processing bottlenecks associated with large-area scalability. Existing consensus stability protocols, such as the International Summit on Organic Photovoltaic Stability (ISOS) procedures, provide a valuable foundation by classifying tests into light-soaking, thermal-cycling, and outdoor-exposure categories. However, these procedures have so far focused primarily on overall device-level power-conversion decay, and have not yet been tailored to specifically monitor interfacial degradation processes, for instance, tracking ion migration across buried interfaces, interfacial reaction-product accumulation, or stress-induced delamination. Moreover, there is an urgent need to establish standardized outdoor testing protocols that explicitly account for the interfacial degradation modes discussed in Sections 2 and 3, including interfacial chemical reactions, mass diffusion, and stress-driven effects, since these processes may evolve differently under diurnal and seasonal cycling than under the continuous stress typical of laboratory accelerated aging. Incorporating such interfacial diagnostic metrics into outdoor testing frameworks will enable a more realistic validation of interfacial engineering strategies, ultimately accelerating the maturation of perovskite photovoltaic technology. On this basis, it is anticipated that the remaining barriers in efficiency, stability, and scalable fabrication can be overcome, propelling single-junction and multi-junction perovskite solar cells from the laboratory to commercialization and providing critical technological support for the global clean energy transition.

## Author contributions

Liangchen Qian and Bohong Chang searched the literature and wrote the manuscript. Danpeng Gao, Bo Li, and Zonglong Zhu edited and modified the manuscript.

## Conflicts of interest

All authors declare no conflicts of interest.

## Data Availability

No original research data, software, or code were included in this review.
